# Networks of Physiological Adjustments and Defenses, and Their Synergy With Sodium (Na^+^) Homeostasis Explain the Hidden Variation for Salinity Tolerance Across the Cultivated *Gossypium hirsutum* Germplasm

**DOI:** 10.3389/fpls.2020.588854

**Published:** 2020-12-08

**Authors:** Kevin R. Cushman, Isaiah C. M. Pabuayon, Lori L. Hinze, Megan E. Sweeney, Benildo G. de los Reyes

**Affiliations:** ^1^Department of Plant and Soil Science, Texas Tech University, Lubbock, TX, United States; ^2^USDA-ARS, Crop Germplasm Research, College Station, TX, United States; ^3^BASF Corporation, Morrisville, NC, United States

**Keywords:** upland cotton, ionic stress, osmotic stress, regulatory network, path analysis, Na^+^ homeostasis, germplasm diversity panel

## Abstract

The abilities to mobilize and/or sequester excess ions within and outside the plant cell are important components of salt-tolerance mechanisms. Mobilization and sequestration of Na^+^ involves three transport systems facilitated by the plasma membrane H^+^/Na^+^ antiporter (SOS1), vacuolar H^+^/Na^+^ antiporter (NHX1), and Na^+^/K^+^ transporter in vascular tissues (HKT1). Many of these mechanisms are conserved across the plant kingdom. While *Gossypium hirsutum* (upland cotton) is significantly more salt-tolerant relative to other crops, the critical factors contributing to the phenotypic variation hidden across the germplasm have not been fully unraveled. In this study, the spatio-temporal patterns of Na^+^ accumulation along with other physiological and biochemical interactions were investigated at different severities of salinity across a meaningful genetic diversity panel across cultivated upland *Gossypium.* The aim was to define the importance of holistic or integrated effects relative to the direct effects of Na^+^ homeostasis mechanisms mediated by *GhHKT1, GhSOS1*, and *GhNHX1.* Multi-dimensional physio-morphometric attributes were investigated in a systems-level context using univariate and multivariate statistics, *randomForest*, and path analysis. Results showed that mobilized or sequestered Na^+^ contributes significantly to the baseline tolerance mechanisms. However, the observed variance in overall tolerance potential across a meaningful diversity panel were more significantly attributed to antioxidant capacity, maintenance of stomatal conductance, chlorophyll content, and divalent cation (Mg^2+^) contents other than Ca^2+^ through a complex interaction with Na^+^ homeostasis. The multi-tier macro-physiological, biochemical and molecular data generated in this study, and the networks of interactions uncovered strongly suggest that a complex physiological and biochemical synergy beyond the first-line-of defense (Na^+^ sequestration and mobilization) accounts for the total phenotypic variance across the primary germplasm of *Gossypium hirsutum*. These findings are consistent with the recently proposed Omnigenic Theory for quantitative traits and should contribute to a modern look at phenotypic selection for salt tolerance in cotton breeding.

## Introduction

High salt concentration in the soil impedes the ability of the roots to extract water, causing dehydration and osmotic stresses with negative impacts to cellular processes that support vegetative and reproductive growth. With prolonged exposure, salt concentration within the plant could build-up leading to cellular toxicity. Injuries are due to physiological perturbations brought largely by cell wall and membrane damage, ionic toxicity and impairment of photosynthesis (Munns, 2002). Adaptive mechanisms for osmotic adjustment, tissue tolerance to accumulated Na^+^ and exclusion of excessive Na^+^ are evolutionarily conserved regardless of the plant’s inherent capacity for tolerance or avoidance (Nakayama et al., 2005; Hauser and Horie, 2010).

Osmotic stress tolerance mechanisms are controlled by genes involved in long distance signaling (*SOS3*, *SnRKs*), osmotic adjustment (*P5CS*, *HKT1*, *SOS1*), and stomatal regulation (*ERA1*, *PP2C*, *AAPK*, *PKS3*) (Berthomieu et al., 2003; Nakayama et al., 2005; Munns and Tester, 2008; Fujii and Zhu, 2012; Amini et al., 2015). In concert, these processes slow down the rate of cell expansion in roots and young leaves (Yeo et al., 1991). Tissue tolerance mechanisms involve the efficient intracellular or intercellular compartmentalization of Na^+^ to prevent toxic effects in the cytoplasm through Na^+^ sequestration into the leaf vacuole by NHX1 and AVP transporters, and exclusion of Na^+^ from cells, xylem and/or roots by HKT1 and SOS1 transporters (Wu et al., 1996; Blumwald et al., 2000; Berthomieu et al., 2003; Foster and Miklavcic, 2019). Exclusion and transport of Na^+^ prevent the rapid build-up of toxicity in the leaves through the regulation of net ion transport to the shoot by SOS3 and SnRK, avoidance of toxic effects in the chloroplast by PP2C and ERA1, reduction of long distance Na^+^ transport by HKT1 and SOS1, and by efficient sequestration of Na^+^ into the root vacuoles. Plants may also alleviate injuries in younger expanding tissues by limiting Na^+^ accumulation in older and less productive basal leaves, which are eliminated by senescence (Cheeseman, 1988; Apse et al., 1999; Blumwald et al., 2000; Halfter et al., 2000; Mäser et al., 2002; Chinnusamy et al., 2004; Haro et al., 2005; Brini et al., 2007). In addition, high intercellular Na^+^ in plants leads to the build-up of reactive oxygen species (ROS) thereby causing oxidative stress, that creates damage to cell membranes, lipids, proteins and nucleic acids (Chakraborty et al., 2016). Peroxidase (PER) and catalase (CAT) are two of several antioxidant enzymes that can minimize tissue damage due to elevated ROS (Mittova et al., 2004; AbdElgawad et al., 2016). The capacity to enhance production of enzymatic and non-enzymatic antioxidants can improve tolerance under salinity stress and protect the photosynthetic machinery (Chakraborty et al., 2016).

Numerous studies in *Arabidopsis* and several crop plants have shown that positive net gains in salinity tolerance can be achieved with the overexpression of critical genes involved in Na^+^ transport and sequestration. For instance, overexpression of *HKT1* has been shown to regulate the vertical distribution of Na^+^ and K^+^, and export of Na^+^ out of the xylem (Møller et al., 2009; Hauser and Horie, 2010). Induction of *HKT1* in the roots and lower leaves has also been shown to reduce Na^+^ concentration in the xylem sap, protecting the younger and more sensitive apical shoot meristems from toxic effects (Sunarpi et al., 2005; Davenport et al., 2007; Zhang et al., 2017; Liu et al., 2019; Sriskantharajah et al., 2020). Additionally, HKT1 has an important role in phloem loading by regulating the removal or recirculation Na^+^ back to the roots (Berthomieu et al., 2003). The plasma membrane-associated SOS1 protein exports Na^+^ to the apoplast and intercellular spaces. It facilitates the removal of Na^+^ in root epithelial cells in association with pores and excretory glands (Shi et al., 2002; Ji et al., 2013; Foster and Miklavcic, 2019). NHX1 sequesters Na^+^ into the vacuole to compartmentalize but not eliminate excessive intracellular levels (Apse et al., 1999; reviewed in Zhang and Shi, 2013).

Relative to most other crop plants, the tetraploid cultivated cotton (*Gossypium hirsutum* L.) has considerably better tolerance to dehydration, osmotic and ionic stresses, hence it is commonly grown in semi-arid and salinity-prone environments (Maas and Hoffman, 1977). Because domestication and subsequent cultivar development were driven primarily by selection for seed yield and fiber-related attributes, it is perceived that limited variability exists across landraces and modern cultivars with respect to physiological traits that enhance the potential for osmotic and/or ionic stress tolerance. Despite this general perception, plant breeders and physiologists are in continuous search for elite cultivars or landraces exhibiting relative superiority in terms of the stress physiological attributes that can be combined with fiber yield and quality traits. A major hurdle is the lack of consensus set of physiological and biochemical parameters that can reveal meaningful variation across the germplasm that are also indicative of differences in overall potentials. Nevertheless, efforts to screen the germplasm for salinity tolerance at the whole plant level using both laboratory and field-based assays have revealed quantifiable inter-genotypic variation (Leidi and Saiz, 1997; Basal et al., 2006; Hemphill et al., 2006).

The *Gossypium* Diversity Reference Set (GDRS) is a germplasm panel representing the spectrum of geographic distribution as well as morphological and allelic diversity across landraces and cultivars collected worldwide (Hinze et al., 2016, 2017). To uncover meaningful variation for salinity tolerance potential across the germplasm, we conducted an extensive study on a representative subset of haplotypes (*core-GDRS*) as minimal comparative panel for physiological attributes relevant to the mechanisms of tolerance or avoidance at the vegetative stage. Our overall findings were consistent with the general observation that the cultivated tetraploid *Gossypium* has relatively high tolerance potential. This was based on generally similar responses observed across cultivars and landraces at relatively moderate level of salinity (EC∼20dS/m) that would be otherwise detrimental to most other plants including Arabidopsis. However, screening at higher levels of salinity (EC > 20dS/m) in hydroponics revealed another layer of variation, which is suggestive of a hidden potential that should be explored further for gene discovery and stress tolerance breeding. An important question that emerged from such hidden potential was the possible contributions of known biochemical and physiological mechanisms that have been uncovered by functional genomics and forward genetics in *Arabidopsis* and few other crop plants.

This study was conducted with the goal of defining at high-resolution, the gradient of salinity tolerance potentials across the *core-GDRS* using an integrative physiological, biochemical and whole plant-level phenomics, aided by network and path analyses. This study aimed to understand the critical interactions that may cause either physiological gains or drags and assess the contributions of the major regulators and facilitators of Na^+^ exclusion and transport mechanisms (vertical and horizontal), namely *GhHKT1, GhSOS1* and *GhNHX1*. Results illuminate the complex but hidden physiological interactions exhibited by the more stress-hardy plant species *Gossypium hirsutum* that may not be revealed by studies using more sensitive model and crop plants. Lastly, this study establishes the foundation for a network-centered discovery paradigm to enhance the precision of phenotypic selection in cotton breeding.

## Materials and Methods

### Evaluation of Salinity Stress Responses Across the Core-GDRS

The comparative panel referred as *core-GDRS* was comprised of twenty-five (25) accessions selected from the US National Cotton Germplasm Collection (College, Station, TX, United States). This subset encompasses eighteen (18) global locations supported with the total range of allelic haplotypes based on 105 polymorphic simple sequence repeat (SSR) loci that capture the greatest proportion of diversity as revealed by PowerCore (Kim et al., 2007; Hinze et al., 2016). Publicly available SSR datasets were used for Principal Coordinate Analysis (PCoA) to correlate the allelic diversity with variation for salinity stress tolerance (Hinze et al., 2017). The minimal comparative panel represents a further reduced subset selected from the *core-GDRS* based on salinity tolerance ranking and included a known salt-sensitive cultivar TX-307 as well as the genome RefSeq genotype TM1 (Li et al., 2015; Zhang et al., 2015).

Salinity stress experiments were conducted under greenhouse conditions at 30–35°C/24–26°C day/night temperature regime, 20% to 30% relative humidity (RH), and 12h photoperiod with 500 μmol m^–2^s^–1^ average light intensity on a customized continuously flowing tube-network hydroponic system with a 130 L reservoir tank (Diversity-D Inc., Brownville, TX, United States). The automated system monitored pH and electrical conductivity (EC) at constant intervals during the experiment. Three parallel hydroponic systems with a total capacity of 60 plants each were used for two (2) salinity stress and one (1) control experiments. Each experiment was comprised of five (5) replicates randomly positioned around the hydroponic tube-networks. The stock solution of the growth medium was comprised of Peter’s Professional Hydroponic Special Fertilizer 5-11-26 (JR Peter’s Inc., Allentown, PA, United States) at full-strength (1 g/L), amended with 0.66g/L CaNO_3_ at pH 6.5 and EC = 2.5dS/m.

Seedlings were first established in standard peat moss potting mix until the fourth node (N4 stage) and then transplanted to the hydroponics system with 1/4 strength media until fully acclimated (N5 stage). Media strength was gradually increased over a one-week period until full-strength. The stock solution was fully drained from the hydroponics reservoir before the addition of the salinized media. The initial stress optimization experiment was conducted using a combination of treatments to create a relatively mild salinity effects. This was achieved by a sequential application to the hydroponics of an input NaCl stock solution of 200mM over a period of seven days, creating a salinized media at EC∼15dS/m. The strength of salinity was subsequently elevated using an input NaCl stock solution of 300mM to create a salinized media at EC∼25dS/m.

The stress treatments that were ultimately applied to all phenotypic comparisons across the diversity panel was designed to create significantly higher levels of stress effects relative to the optimization experiments. These experiments were designed to reveal hidden components of the total phenotypic variance that could not be revealed by the salinity effects used in the optimization experiments. The salinity treatments involved the sequential application of increasing NaCl strength to the hydroponics, *i.e.*, low concentration (mild effects) then medium concentration (moderate effects) and finally high concentration (severe effects). For the low concentration, the hydroponics system was injected with an input stock solution of 250 mM NaCl, which created an EC∼20dS/m when diluted in the hydroponics media. Plants were kept under this condition for seven days. To further reiterate the stress effects of the prolonged exposure to EC∼20dS/m, the salinity was further elevated to medium and high strength by sequentially injecting an input stock solution of 500 mM and 750 mM NaCl to establish the salinized hydroponics at EC∼40dS/m and EC∼58dS/m, respectively, over a much shorter time interval of three days. Progression of visible symptoms of injuries was observed under this experimental set-up for the next few days.

### Standard Evaluation of Salinity Stress Injury

After three days at EC∼58dS/m, the overall health status and vigor of each plant were assessed qualitatively by assigning a *Standard Evaluation Score* (SES) using a modified scale of 0 to 10 with decreasing severity of stress injury (Gregorio et al., 1997). SES scoring was performed blindly with each plant referenced according to their positions in the hydroponics matrix. Based on the overall distribution of SES, accessions in the comparative panel were classified as very sensitive/inferior (SES < 2), sensitive (4.0 < SES > 2.0), moderately tolerant/intermediate (5.0 < SES > 4.0), tolerant (6.0 < SES > 5.0), and very tolerant (SES > 6.0). SES were averaged across experiments and replicates (n = 5) to generate a mean for each genotype.

### Measurement of Growth and Physiological Parameters

**FIGURE 1:**
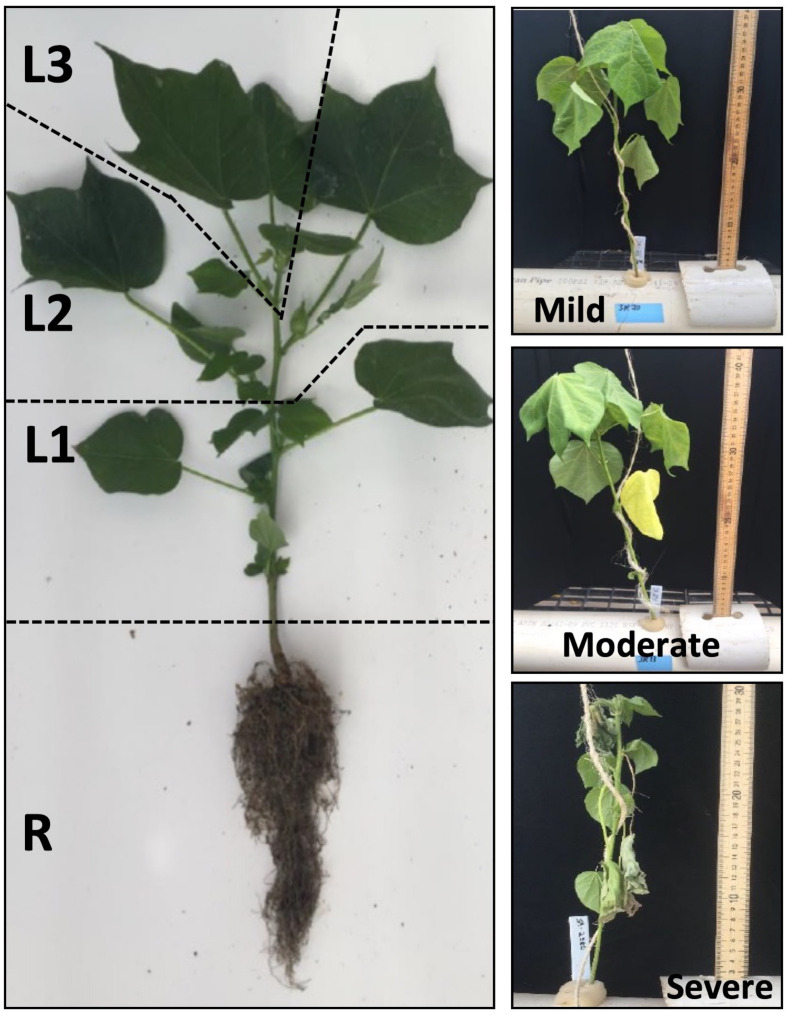
Spatial sampling scheme for the analysis of the vertical distribution of Na^+^ and K^+^, and for profiling the expression of the major genes involved in Na^+^ homeostasis (*GhHKT1*, *GhSOS1*, *GhNHX1*) at mild, moderate and severe salinity. The four sampled positions along the vertical axis were designated R (Roots), L1 (oldest leaves in the lowest zone of the shoot axis; least sensitive), L2 (mid-age leaves in the middle zone of the shoot axis; moderately sensitive), and L3 (youngest leaves in the uppermost zone of the shoot axis; most sensitive).

The physiological status of each accession in the comparative panel was evaluated using multiple parameters at 24, 96 and 168 h after exposure to the progressive salinity treatments. Shoot length (*L*) was measured from time-0 (*t*_0_) to time-n (*t*_n_) under control and stress conditions. Chlorophyll content was measured with the MC-100 Chlorophyll Concentration Meter (Apogee Instruments, Utah, United States) and expressed as chlorophyll concentration in μ*mol m^–2^*. The chlorophyll meter was calibrated with a blank each time the meter was turned on and set to the default settings of the manufacturer. Leaf chlorophyll content was measured twice along the vertical shoot axis at positions L1 (N1to N2 stage), L2 (N3 to N4 stage), and L3 (≥ N5 stages) with three (3) replicates across three parallel hydroponics system (Figure 1). Stomatal conductance was measured with the SC-1 Leaf Porometer (Meter Group, Inc., Pullman, WA, United States) with single measurement per leaf with three repeats. Porometers were calibrated according to the daily greenhouse conditions and set to the manufacturer’s default settings (Supplementary Dataset 1). At each time point, three plants per genotype (*n* = 3) were harvested and fresh weight (FW) was determined for roots and shoots. Dry weights (DW) were determined by drying the roots and shoots at 50°C for 7 days. Water content (%) was calculated by *(FW-DW)/FW*^∗^100 (Liu et al., 2019). Since plant material was limited, water content was used to estimate the biomass content at EC∼20dS/m and EC∼40dS/m.

### Temporal Tissue Sampling and Electrolyte Leakage Analysis

For all chemical analysis and biochemical assays, plants were sampled at four positions along the vertical axis of the plant, representing different organs (R = roots, L = shoot/leaves) and/or developmental age of shoot organs (L1 = oldest leaves/most insensitive, L2 = mid-age leaves/intermediate, L3 = youngest leaves/shoots/most sensitive) (Figure 1). For the electrolyte leakage (EL) analysis, 5 mm leaf disks were collected at L1, L2 and L3 for a total of three (3) plants per genotype. The %EL was determined by measuring the EC in 2 ml nanopure water (16 to18 MΩ) before and after stress using a Fisherbrand^TM^ Traceable^TM^ conductivity meter (Thermo Scientific, Waltham, MA, United States) (Ballou et al., 2007). The %EL from intact tissues was determined relative to total tissue electrolytes after boiling and expressed either as %EL or electrolyte leakage index (ELI) (de los Reyes et al., 2013).

### Tissue Peroxide Content, and Total Peroxidase and Catalase Activities

Total peroxide (PRX) content and total peroxidase activity were determined using the Amplex^®^ Red Hydrogen Peroxide/Peroxidase Assay Kit (Invitrogen, Carlsbad, CA, United States; de los Reyes et al., 2013). Briefly, 50 mg and 25 mg tissues were used for PRX and PER extraction in 500 μL and 1000 μL of 20 mM sodium phosphate (Na_3_PO_4_) buffer at pH 6.5. The modified assay was performed with 300 μL of 0.2 U/mL peroxidase, 120 μL of 100 μM Amplex^®^ Red/DMSO solution, and 18 mL of 1X reaction buffer for PRX determination, and 1500 μL of 2 mM H_2_O_2_, 120 μL of 100 μM Amplex^®^ Red/DMSO solution, and 16 mL of 1X reaction buffer for PER activity measurements. Assays in 50 μL volume were performed in 96-well microplates with absorbance measurements at 560 nm on an iMark^TM^ Microplate Reader (Bio-Rad, Hercules, CA, United States). Total catalase activity was determined using the Amplex^®^ Red Catalase Assay Kit (Invitrogen, Carlsbad, CA, United States) according to manufacturer’s protocols. Due to high CAT activities especially on the salt stressed plants, 60 μL H_2_O_2_ was used for the initial reaction with absorbance measurements at 560 nm. Total PER and CAT activities were extrapolated from standard curves with *R*^2^ > 0.97.

### Lipid Peroxidation Assay

Samples were obtained from R, L1, L2 and L3 tissues (100 mg) and extracted with 1.75 mL of 0.1% (w/v) trichloroacetic acid (TCA) by centrifugation at 10,000 *g* at 20°C for 15 min. The supernatant (375 μL) was incubated with 750 μL of 20% (w/v) TCA and 750 μL of 0.5% (w/v) thiobarbituric acid (TBA) for 30 min at 95°C (Jambunathan, 2010). Absorbance of the cooled reaction mixtures (200 μL) was determined at 530 nm and 600 nm. The total lipid peroxidation products was calculated using the Beer-Lambert equation: *C* = *A/(*ε × *l)*, where *A* is the difference in absorbance at 530–600nm, ε is the extinction coefficient of 155 mM-1cm^–1^, *C* is lipid peroxide concentration in mM, and *l* is the length of the cuvette in cm.

### Total Antioxidant Capacity Assay

The DPPH (2,2 diphenyl-1-picylhydrazil) method was used to determine the total antioxidant capacity in the R, L1, L2 and L3 samples (Prakash et al., 2001). Briefly, 100 mg of ground tissues were extracted with 500 μL absolute ethanol. The assay solution contained 1 mg of DPPH per 6 mL of absolute ethanol, combined with 100 μL of the plant extract. Absorbance at 520 nm was determined after 10 min incubation in microplates. For the standard curve, 4 mg/50 mL of L-ascorbic acid was used as the starting solution. The radical scavenging activity was calculated by: *[1-(Abs_*sample*_/Abs_*control*_)]* × *100*.

### Determination of Proline Content

A procedure modified from Bates et al. (1973) was used to determine the proline (PRO) concentration in R, L1, L2 and L3 samples. Briefly, 100 mg of pulverized tissues were extracted in 500 μL 3% (w/v) sulfosalicylic acid. The assay solution contained 1.25 g ninhydrin, 80 mL glacial acetic acid, 20 mL 6 M phosphoric acid, and 25 mL 3% (w/v) sulfosalicylic acid. Supernatant from the extraction mixture (100 μL) was combined with 500 μL of assay solution and incubated in 95°C water bath for an hour followed by ice-quenching, and addition of 1 mL of toluene. After fractionation, absorbance of 100 μL organic phase was determined at 520 nm in 96-well microplate, with standard curve of 200 μM increments of L-proline.

### Determination of Ion Content of Plant Tissues

A total of five (5) plants for each genotype were sampled at R, L1, L2 and L3. Tissue samples were collected 24 h after each three-day incremental increase in NaCl input, and then oven-dried at 50°C for 7 days. Dried tissues (1 g) were pulverized and analyzed for Na^+^, K^+^ content as well as nine (9) other elements through the standard nitric-perchloric acid digestion method, measured on AA unit per Western States Ver 4.00, P-4-20 (AOAC, 1990; A and L Plains Analytical Laboratory, Lubbock, TX, United States). Of the total nine elements investigated, only four (4) others in addition to Na^+^ and K^+^, *i.e.*, Mg^2+^, Ca^2+^, Mn^+2^, Fe^3+^, showed fluctuations due to stress (Supplementary Dataset 1).

### Identification of the *Gossypium* Na^+^ Homeostasis Genes

BLASTP queries with segments of protein sequences from *GhHKT1, GhSOS1*, and *GhNHX1* were performed against published genome sequences to identify potential orthologs. The cDNA sequences for the target genes were imported into Geneious 6.1.6. (Biomatters Ltd., Auckland, New Zealand), and aligned using ClustalW. Sequences were trimmed to adjust to comparable lengths (Supplementary Datasets 2–4, available at https://doi.org/10.5061/dryad.6wwpzgmwd). Maximum likelihood (ML) analysis was performed with the RAxML method using the online CIPRES portal with 1,000 bootstrap replicates (Stamatakis et al., 2008; Miller et al., 2010). Maximum parsimony (MP) analysis was performed with PAUP^∗^ 4.0b10, and branch support was assessed with 1,000 non-parametric bootstrap replicates (Felsenstein, 1985; Swofford, 2002). Orthology of gene loci was inferred when sequences were monophyletic within the genus *Gossypium*. Genes from *G. hirsutum* that were monophyletic with *G. arboreum* were inferred to have originated from the A-subgenome, while genes that were monophyletic with *G. raimodii* were inferred to have originated from the D-subgenome.

### RNA Extraction and Transcript Abundance Analysis by qRT-PCR

Tissue sampling for the extraction of total RNA was according to the same spatial design used in all chemical analysis and biochemical assays (R-L1-L2-L3). Samples were frozen in liquid nitrogen and total RNA was extracted with the Spectrum Plant Total RNA Kit (Sigma, St. Louis, MO, United States) according to the manufacturer’s protocols. The cDNA synthesis was performed with 1 μg of total RNA using the iScript cDNA synthesis kit (Bio-Rad, Hercules, CA, United States). Gene-specific primers for qRT-PCR were designed based on *G. hirsutum*, *G. arboreum*, and *G. raimodii* sequences with the closest homology to the Arabidopsis *AtSOS1, AtNHX1*, and *AtHKT1* as well as other eudicot species. Primer-BLAST was used to design specific primers for each homologous *Gossypium* open reading frames which were validated against the annotated *Gossypium* reference genome. Primer sequences, reference genes, and qRT-PCR conditions are described in Supplementary Table 1. The qRT-PCR assay was performed using the SsoFast EvaGreen Supermix (Bio-Rad) in the CFX384 Real-time PCR system with three biological and two technical replicates. Relative gene expression was calculated by the ΔΔCt method and normalized using ln(x-1) (Schmittgen and Livak, 2008).

### Severed-Phloem and Na^+^ Recirculation Experiments

The severed-phloem method was used to investigate the amount of Na^+^ recirculation back to the roots from the shoots. This experiment was performed with SA-1766 representing the most tolerant genotype and SA-0033 representing the most sensitive genotype. The experiment was performed by girdling the stem 2cm above the crown (Kong et al., 2012). Roots and pooled shoots of control, control-girdled, stressed, and stressed-girdled plants were sampled at EC∼20dS/m and EC∼40dS/m treatments, and tested for stress effect, girdling effect, and genotypic effect on Na^+^ accumulation. Analysis of tissue Na^+^ and K^+^ content was performed using the standard nitric-perchloric acid digestion method (A and L Plains Analytical Laboratory, Lubbock, TX, United States).

### Statistical Analysis, randomForest, and Path Analysis

All statistical analyses were conducted with R 3.5.2 (R Core Team 2013). Salt-tolerance indices were calculated by dividing the individual stress parameters with the corresponding means of control parameters (Munns, 2002). Individual variables were tested for normality using the Shapiro-Wilks test, and the variables that were highly skewed were transformed to either log or square root scales. Datasets were normalized for univariate or multivariate analyses. Tukey’s test (Agricolae Package) was used for multiple comparison of means (de Mendiburu, 2010). Multivariate normality was also tested using the MVN package (Korkmaz et al., 2014). Individual parameters were transformed for normal distribution.

Principal component analysis (PCA) was performed using the *prcomp* function to investigate the relationship of multiple physiological, chemical, and biochemical properties. Eigenvectors were displayed on the ordination using the *envfit* function in the ‘Vegan’ Package with a significance cut-off at *p* = 0.05, and magnitude of the vector indicating significance (Oksanen et al., 2019). The importance of variables was determined by ‘*randomForest’ Package* in R that made use of 1/3 of the data matrix for model training with 1,000 replicates (Liaw and Wiener, 2002). Random forest classified different objects into groups through a machine learning regression tree algorithm, which assesses the relative importance of multiple variables contributing to a trait and their interactions through an iterative process. Random forest weighed the importance of each variable in a set of classifications such SES. Quantitative variables were scaled to have equal means and variances. To facilitate the model training, the classification was reduced to the most tolerant, neutral and most sensitive categories with four accessions in each category. The importance of variables was calculated using the ‘*importance*’ function on the ‘*randomForest’* results.

To investigate the interaction between variables, theoretical models were created for potential interactions collectively driven by the empirical data generated and other information from the literature (Supplementary Table 2). Each model consists of squares that represent measured physiological, biochemical or molecular attribute connected by arrows. The direction of the arrows represents either a positive or negative causal relationship, with the two-sided arrows representing the co-varying interactions. Models were tested using path analysis with ‘*Lavaan’ Package* in R, which calculated multiple regressions simultaneously while taking into account the latent or unmeasured variable to assess the significance of each interaction and goodness of fit of the model (Rosseel, 2012). The general classes or families of physiological outcomes that can be derived from the integration of various variables were *Ion Transport and Homeostasis* (including Na^+^ and K^+^), *Radical Scavenging and Oxidative Defenses*, and *Photosynthesis and Metabolism*. To achieve the appropriate sample size (*n* = 144) for each class, data from L1, L2, and L3 were pooled from each time point or stress level for total shoot measurements. All root data across each time point were pooled. Models were generated using the *sem* function on the covariance matrix of transformed and normalized variables.

## Results

### Salinity Tolerance Potential Relative to Genetic Diversity

Previous efforts to compare salinity stress responses across different subsets of non-GDRS and GDRS accessions made use of 200mM to 300mM as input concentrations of NaCl in hydroponics (Leidi and Saiz, 1997; Zhang et al., 2014; Peng et al., 2016). Our preliminary studies on a subset of test germplasm revealed that such NaCl levels imposed only mild stress that did not elicit obvious differential reactions across cultivars at the whole-plant level. After one-week exposure, no significant differences across genotypes could be detected based on key growth parameters (Supplementary Figure 2). However, with sequentially increasing strength of salinity (*i.e.*, from EC∼20 dS/m to EC∼40 dS/m to EC∼58 dS/m) across a much wider subset of core-GDRS accessions, significant variation across the diversity panel (differential response) was revealed. Genotypic differences were revealed based on persistence, relative to the duration and intensity of salinity stress.

**FIGURE 2:**
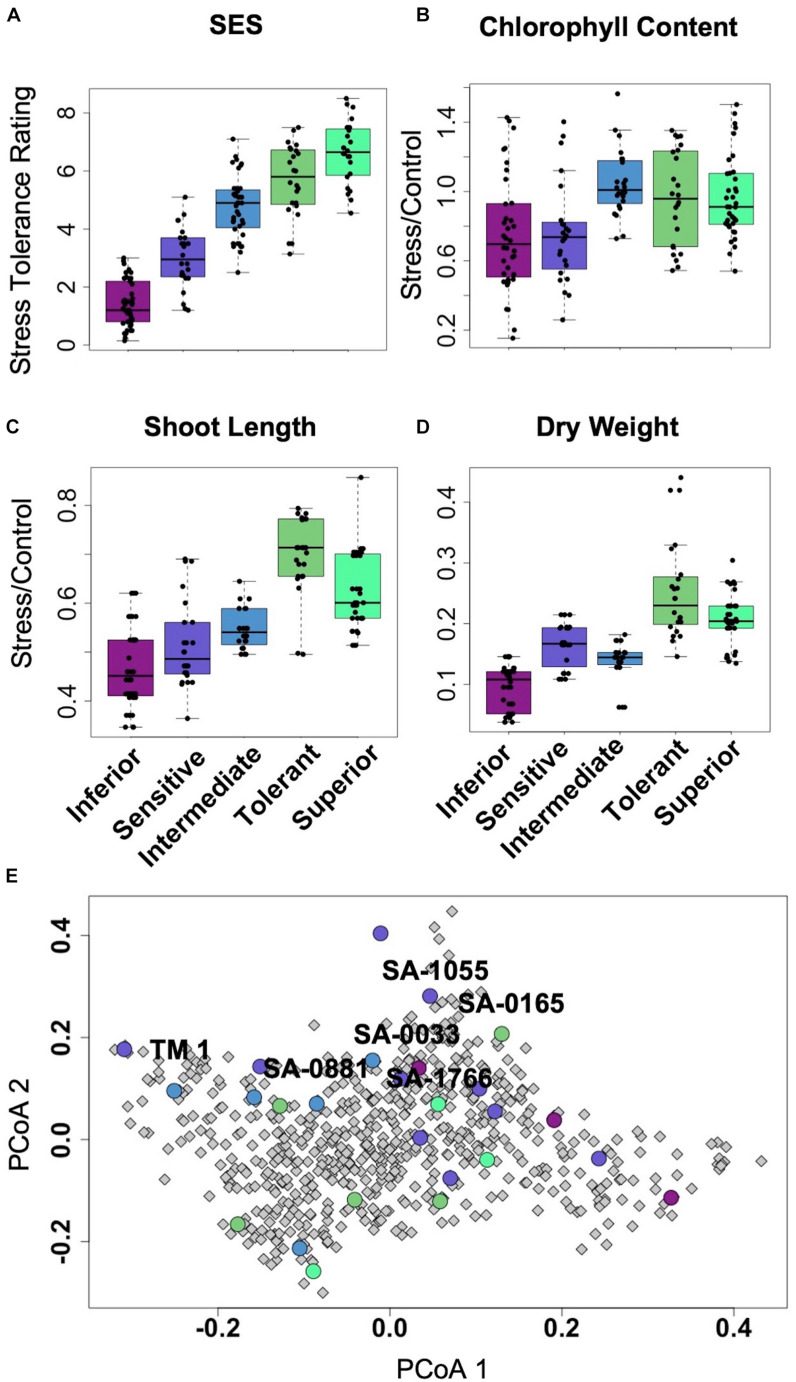
**(A–D)**, Boxplots showing significant differences in growth and health parameters with correspondence to the overall stress tolerance potential as represented by Standard Evaluation Score (SES). Each category across the entire gradient of stress tolerance included at least two genotypes with three to six biological replicates per genotype. **(E)**, Principal coordinate plot of the genetic diversity captured by 105 SSR markers across 655 accessions of improved *G. hirsutum* cultivars and landraces, reconstructed based on salt stress phenotypes and diversity data (Hinze et al., 2016, 2017). The twenty-five (25) homozygous accessions of the core-GDRS plus the genome RefSeq TM1 selected for screening are highlighted (circles) with their respective SES and other growth and physiological parameters.

Of the representative germplasm panel, which included twenty five (25) uncharacterized core-GDRS accessions and two (2) known salt-sensitive controls (TX-307 and the genome RefSeq TM1), a total of twelve (12) accessions appeared to cover the range of stress tolerance potentials relative to the extent of genetic diversity established by SSR-based phylogenetic studies (Figures 2A–D; Table 1; Supplementary Figure 1; Supplementary Dataset 1; Hinze et al., 2017). These accessions were chosen to represent the *minimal comparative panel* for all subsequent physiological analyses. From this panel, the accessions SA-0033 (very sensitive/inferior), SA-1055 (sensitive), SA-0881 (moderately tolerant/intermediate), SA-0165 (tolerant), and SA-1766 (very tolerant/superior) were chosen to represent the reference haplotypes for each step in the phenotypic gradient. In the SSR-based allelic diversity plot, the tolerant and sensitive genotypes appeared to have distinct origins, with the genome RefSeq cultivar TM1 being quite distant from the superior core-GDRS accession SA-1766, and from the other reference haplotypes across the phenotypic gradient (Figure 2E). We hypothesized that the minimal comparative panel including the reference haplotypes meaningfully represent various assemblages of positive and negative attributes that may be influencing the observed variation in tolerance potentials.

**TABLE 1:** Summary profiles of the twelve (12) selected core-GDRS accessions with their tolerance ranking and associated range of SES.

Accession	Tolerance	SES	Chlorophyll Index	Shoot Length Index	Dry Weight Shoots Index
SA-0033	Very Sensitive/Inferior	1.65 (±) 0.84	0.78 (±) 0.33^abc^	0.57 (±) 0.04^b^	0.07 (±) 0.04^f^
SA-2580	Very Sensitive/Inferior	1.63 (±) 0.79	0.86 (±) 0.27^abc^	0.44 (±) 0.06^de^	0.08 (±) 0.02^f^
TX-307	Very Sensitive/Inferior	1.26 (±) 0.68	0.57 (±) 0.06^bc^	0.40 (±) 0.04^de^	0.12 (±) 0.004^ef^
SA-1055	Sensitive	2.23 (±) 1.05	0.45 (±) 0.14^c^	0.44 (±) 0.04^b^	0.14 (±) 0.03^e^
SA-0002	Sensitive	3.10 (±) 1.08	0.73 (±) 0.09^abc^	0.47 (±) 0.03^cd^	0.15 (±) 0.03^de^
SA-0881	Intermediate	4.58 (±) 1.04	0.99 (±) 0.08^ab^	0.55 (±) 0.04^b^	0.14 (±) 0.01^e^
SA-1512	Intermediate	4.84 (±) 1.00	0.95 (±) 0.08^ab^	0.52 (±) 0.06^bc^	0.15 (±) 0.^05cde^
SA-1759	Tolerant	5.72 (±) 1.27	0.94 (±) 0.03^ab^	0.72 (±) 0.05^a^	0.22 (±) 0.03^ab^
SA-0165	Tolerant	5.63 (±)0.95	1.01 (±) 0.33^a^	0.68 (±) 0.10^a^	0.20 (±) 0.03^bcd^
SA-1766	Very Tolerant/Superior	6.90 (±) 1.16	0.82 (±) 0.01^abc^	0.58 (±) 0.04^b^	0.20 (±) 0.005^bc^
SA-2895	Very Tolerant/Superior	6.87 (±) 0.82	0.92 (±) 0.18^ab^	0.72 (±) 0.04^a^	0.21 (±) 0.06^ab^
SA-3284	Very Tolerant/Superior	5.89 (±) 1.16	0.97 (±) 0.17^ab^	0.58 (±) 0.05^b^	0.26 (±) 0.07^a^

*The visible physiological parameters that partially contributed to SES are listed with ranges. Group assignment made by Tukey’s HSD are designated by letter group. Indices are salt stress values divided by their control value.*

### Physiological and Biochemical Properties Contributing to Phenotypic Gradient

Principal component analysis (PCA) was performed to investigate if the relative stress tolerance ranking across the core-GDRS could be supported by the inherent variation for other physiological and biochemical properties. This analysis was performed using the spatio-temporal profiles for stomatal conductance, membrane injury as revealed by tissue electrolyte leakage index (ELI), lipid peroxidation (LP), tissue Na^+^, K^+^, Mg^2+^, Mn^2+^, Fe^3+^ and Ca^2+^ contents, proline content (Pro), chlorophyll content (Cp), total peroxide content (PRX), total antioxidant capacity (DPPH), and total catalase (CAT) and peroxidase (PER) activities.

**FIGURE 3:**
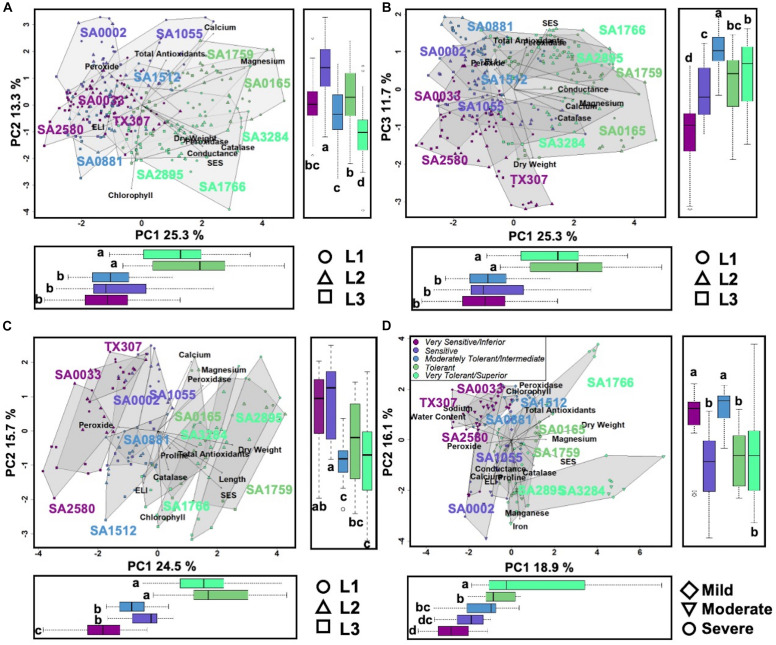
**(A)** and **(B)**, Scatterplots of shoot PCA at mild to moderate salinity. PC1 explained 25.3% of the variation, which significantly differentiates the tolerant and superior genotypes (*R*^2^ = 0.505, *p* < 0.001). PC2 explained 13.3% of the variation, which significantly differentiates the superior genotypes (*R*^2^ = 0.46, *p* < 0.001). PC3 explained 11.7% of the variation, which significantly differentiates the inferior genotypes (*R*^2^ = 0.40, *p* < 0.001). Symbols in the lower right of the panel indicate leaf position. **(C)**, Scatterplots of shoot PCA at severe salinity. PC1 explained 24.5% of the variation, which significantly differentiates the tolerant and superior genotypes (*R*^2^ = 0.68, *p* < 0.001). PC2 explained 15.7% of the variation, which significantly differentiates the inferior and sensitive genotypes (*R*^2^ = 0.33, *p* < 0.001), and it also significantly differentiates the leaf positions mostly with L3 (*R*^2^ = 0.35, *p* < 0.001). **(D)**, Scatterplots of root PCA at mild to severe salinity. PC1 explained 18.9% of the variation, which significantly differentiates the genotypes (*R*^2^ = 0.50, *p* < 0.001). PC2 explained 16.1% of the variation, which significantly differentiates the inferior (*R*^2^ = 0.26, *p* < 0.001). Symbols in the lower left indicate the three EC levels. Boxplots on the bottom and side panels for all figures exhibit the significant differences in the clusters. Letters indicate groups assigned by Tukey pairwise comparisons.

Integration of the profiles along the vertical leaf/shoot axis of the plant, *i.e., L1* = *oldest/least sensitive, L2* = *mid-age/intermediate, L3* = *shoot/most sensitive* (Figure 1), for all physiological, chemical and biochemical parameters showed that at low (EC∼20dS/m) to medium (EC∼40dS/m) strength of input NaCl to the hydroponics, three principal components explained 50.3% of the total phenotypic variance (Figures 3A,B). The superior and tolerant genotypes (including SA-1766 and SA-0165) were significantly separated from the inferior and sensitive (including SA-0033 and SA-1055) and intermediate (including SA-0881) genotypes along PC1, which explained 25.3% of the total variance. The driving eigenvectors along this axis that also correlated with SES were stomatal conductance, Mg^2+^ content, dry weight, and total catalase and peroxidase activities, with negative contributions from PRX, and membrane integrity as measured by ELI. The PC2 and PC3 explained 13.3% and 11.7% of the total variance, respectively. The PC2 separated the rest of the genotypes from the superior group based mainly on total chlorophyll content, Ca^2+^ content, and total antioxidants, while the PC3 separated the inferior genotypes by virtue of the SES, PRX, total antioxidants, and dry weight.

With much higher input NaCl (EC∼40dS/m to EC∼58dS/m) to the hydroponics, the L1-L2-L3 profiles of superior and tolerant genotypes were significantly different from the inferior, sensitive, and intermediate genotypes along PC1, which explained 24.5% of the total variance (Figure 3C). The major attributes driving this separation are SES, relative shoot length, dry weight, total antioxidants, and PRX. The PC2 significantly separated the inferior and sensitive genotypes from the intermediate, tolerant, and superior genotypes with 15.7% of the phenotypic variance. Variation within genotype relative organ positions (L1-L2-L3 profiles) was also significantly correlated with this axis.

In the roots (R) under all levels of stress, PC1 significantly separated the tolerant and superior genotypes from the other phenotypic classes, explaining 18.9% of the phenotypic variance (Figure 3D). The most critical contributors to this axis are SES, tissue Mg^2+^ and Na^+^ contents, dry weight, PRX, and water content. In addition, much of the variation among individuals within the tolerant phenotypic class occurred on this axis and dispersed by the severity of salt stress. PC2 explained 16.1% of phenotypic variance and segregated the genotypes belonging to the tolerant classes from the majority of the sensitive genotypes. Tissue Mn^2+^ and Fe^3+^ contents, PER, and total antioxidants are major contributors to this axis.

### Spatio-Temporal Patterns of Na^+^ and K^+^ Accumulation

Root uptake of Na^+^ may occur either through non-selective cation transporters, non-discriminating K^+^ transporters or both. Absorbed Na^+^ can be extruded externally via Na^+^/H^+^ antiporters. The capacity for balancing uptake, extrusion, xylem unloading, and intercellular and intracellular mobilization across less sensitive (L1) to more sensitive (L3) organs are inherent properties whose importance in tolerance have been established. To address the potential contribution of these mechanisms to the observed phenotypic gradient across the core-GDRS, Na^+^ accumulation profiles were compared across the minimal comparative panel. For clarity we only compared the most tolerant and most sensitive classes.

**FIGURE 4:**
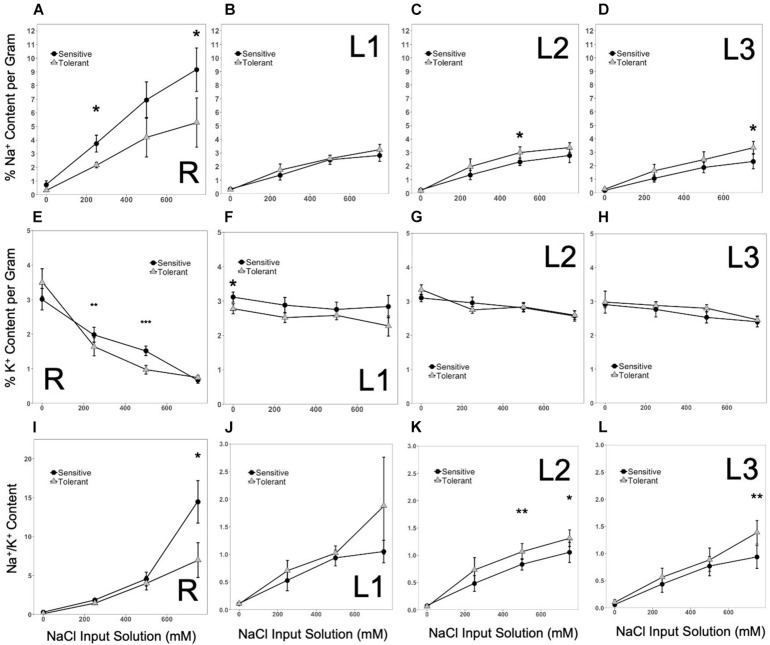
**(A–D)**, Patterns of Na^+^ accumulation in tissues at increasing input NaCl along the vertical axis of the plant (R, L1, L2, L3, respectively). Significant increases in Na^+^ concentrations occurred in sensitive plants over time. In the shoots, Na^+^ concentrations increased in tolerant genotypes. Na^+^ increased first in the lower and middle shoots, but as the stress intensified, the sensitive upper shoot accumulated comparable levels of Na^+^ (*significant at *p* < 0.05). **(E–H),** Patterns of K^+^ accumulation in tissues at increasing input NaCl along the vertical axis of the plant (R, L1, L2, L3, respectively). Significant decreases in K^+^ concentrations occurred in the roots as the stress intensified, which was initially more pronounced in tolerant genotypes. In the shoots, K^+^ concentrations remained constant across genotypes (significant at *p < 0.05, **p < 0.01, and ***p < 0.001). **(I–L)**, Patterns in Na^+^/K^+^ in tissues at increasing input NaCl along the vertical axis of the plant (R, L1, L2, L3, respectively). Significant increases occurred in the Na^+^/K^+^ in roots of sensitive plants over time. In the shoots, Na^+^/K^+^ increased in tolerant genotypes (significant at *p < 0.05 and **p < 0.01). For clarity only the most tolerant and most sensitive classes are shown. Error bars indicate standard error.

With increasing concentrations of input NaCl in the hydroponics from mild to moderate to harsh stress, the total Na^+^ content in both roots (R) and shoot axis/leaves (L1-L2-L3) also increased (Figures 4A–D). The root (R) profiles indicated a slightly higher Na^+^ uptake in the inferior genotypes at all levels of NaCl input than the superior genotypes (Figure 4A). This trend suggests that superior genotypes may have higher capacities to extrude excessive Na^+^ absorbed by the roots, presumably through mechanisms that may involve Na^+^/H^+^ antiporter systems. One peculiar trend observed in the L1-L2-L3 profiles of Na^+^ accumulation was the reverse pattern between inferior and superior genotypes. While this trend appeared to be contradictory to the lower rate of Na^+^ uptake by the roots in superior genotypes, the earlier onset of senescence observed in older L1 leaves of inferior genotypes may be a contributing mechanism that somehow delays the upward movement and distribution of Na^+^ to the more fragile mid-age L2 leaves (Figures 4B,C). However, with progressive increase in input NaCl, the rate of Na^+^ accumulation became more comparable between superior and inferior genotypes. The exception was in the youngest L3 leaves, where the rate of accumulation remained constant in the superior genotypes while tailing off in the inferior genotype (Figure 4D). Overall, these trends suggest that while root Na^+^ uptake is relatively lower in superior genotypes, the higher Na^+^ accumulation up to the mid-age L2 leaves in superior genotypes indicates that the threshold of sensitivity to Na^+^ toxicity is much lower among inferior genotypes. Thus, lower levels of Na^+^ caused cellular injuries in inferior genotypes while revealing different patterns of spatial Na^+^ accumulation in the superior genotypes. The profiles of K^+^ accumulation in the L1-L2-L3 axis showed no significant changes across stress levels, genotypes or organ positions (Figures 4F–H). The K^+^ uptake profiles in the roots (R) showed a significant decline with increasing strength of NaCl input, which appeared to be accelerated in superior genotypes (Figure 4E). Given the flat trend in shoot K^+^ accumulation, increases in Na^+^/K^+^ ratios were essentially determined by Na^+^ accumulation profiles (Figures 4I–L).

### Differential Expression of *HKT1* Genes

**FIGURE 5:**
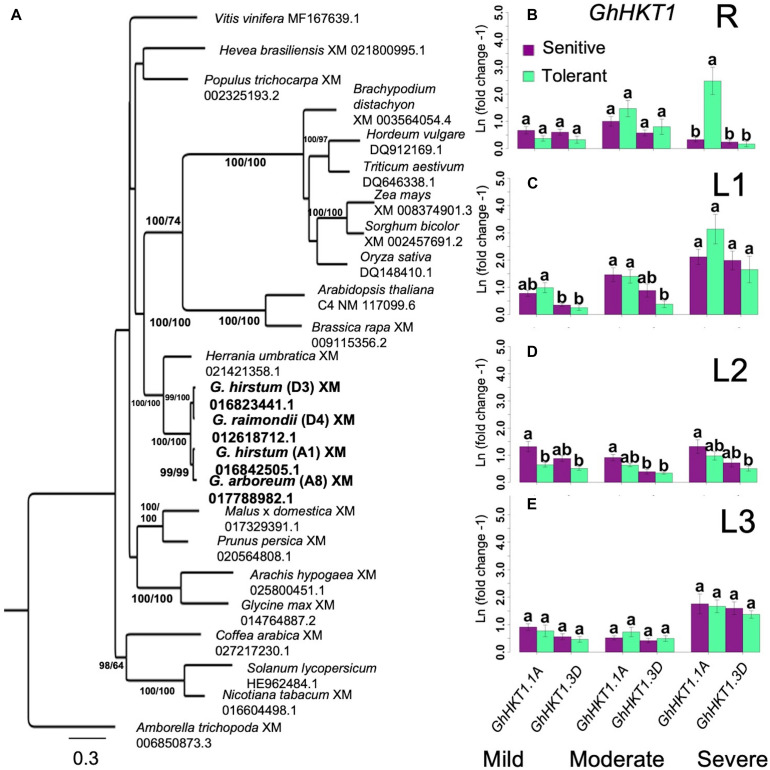
Analysis of *HKT1* homology and expression. **(A)**, Identification of orthologous *GhHKT1.* Bootstrap values are Maximum Likelihood (ML)/Maximum Parsimony (MP). Consistency index (CI) = 0.489; Retention index (RI) = 0.4644. **(B–E)**, Spatial expression profiles of orthologous *GhHKT1* genes in sensitive and tolerant genotypes along the vertical axis (**B** = roots/R; **C** = leaves/L1; **D** = leaves/L2; **E** = shoots/L3). Gene expression tends to increase with increasing severity of stress. For the shoots, no significant difference in transcript abundance associated with tolerance or subgenomic origin of orthologs is evident. In the roots at the highest stress level, there was a significant upregulation of the A-subgenome ortholog (groups assigned by Tukey pairwise comparisons). For clarity only the most tolerant and most sensitive classes are shown. Error bars indicate standard error.

Based on studies in Arabidopsis, one important function of HKT-type transporters is to facilitate Na^+^ influx to the roots and its recirculation in the phloem (Apse and Blumwald, 2007; Davenport et al., 2007). Given the contrasting profiles of root Na^+^ uptake and shoot axis/leaf Na^+^ accumulation between superior and inferior genotypes (Figures 4A–D), the spatio-temporal expression *GhHKT1* was compared between the extreme classes in order to assess their importance to the observed physiological variation. BLAST queries identified two potential orthologs of the Arabidopsis *AtHKT1* in the tetraploid cultivated *G. hirsutum*, one located on chromosome-1 (A1 in A-subgenome) hence *GhHKT1.A1*, and the other on chromosome-16 (D3 in D-subgenome) hence *GhHKT1.D3*. Each of these orthologs formed monophyletic clades with each of the progenitor diploid *G. raimondii* Ulbr. and *G. arboreum* L. genomes at 99% bootstrap support that corresponded to the appropriate subgenome (Figure 5A). Together, the *GhHKT1* loci of the three *Gossypium* species were monophyletic with 100% bootstrap support, sister to its closest taxa *Herrania umbratica* (R. E. Schult).

Under mild stress, both *GhHKT1.A1* and *GhHKT1.D3* showed slight to moderate downregulation in roots (R) in both the inferior and superior genotypes, which could be an effect of osmotic shock. With an elevated level of Na^+^ input to impose moderate stress, expression of both *GhHKT1.A1* and *GhHKT1.D3* increased to nearly the control levels but still without significant difference between inferior and superior genotypes. Further incremental increase in NaCl input to harsh stress caused significant upregulation of *GhHKT1.A1* only in the superior genotypes, but not *GhHKT1.D3* whose expression remained around the control level (Figure 5B). While the superior genotypes appeared to exhibit a unique signature of *GhHKT1.A1* upregulation under high salt, such pattern correlated with increased translocation to the shoots but not Na^+^ accumulation in the roots in the tolerant genotypes, as indicated by tissue Na^+^ content (Figure 4A) and Na^+^/K^+^ ratio (Figure 4I). This trend suggests that novel combinations of transport mechanisms could be largely responsible for the observed variation in root Na^+^ uptake across the core-GDRS.

Na^+^ is transported and distributed vertically to the shoot upon absorption through the roots. The temporal expression patterns of *GhHKT1* genes between extreme genotypes showed that mild to moderate stress levels did not affect the expression of *GhHKT1.A1* nor *GhHKT1.D3* in the leaves, regardless of developmental age and position along the L1-L2-L3 axis in both the inferior and superior genotypes. However, both *GhHKT1* orthologs were significantly upregulated under severe stress in sensitive and tolerant genotypes (Figures 5C–E). While HKT-type transporters are known to play some roles in regulating Na^+^ allocation between roots and shoots (Apse and Blumwald, 2007; Davenport et al., 2007), based on the gene expression patterns along the L1-L2-L3 axis and the lack of direct correlation with Na^+^ accumulation profiles (Figures 4B–D), the precise contributions of *GhHKT1.A1* and *GhHKT1.D3* in inferior and superior cultivars is not clear.

### Differential Expression of *SOS1* Genes

**FIGURE 6:**
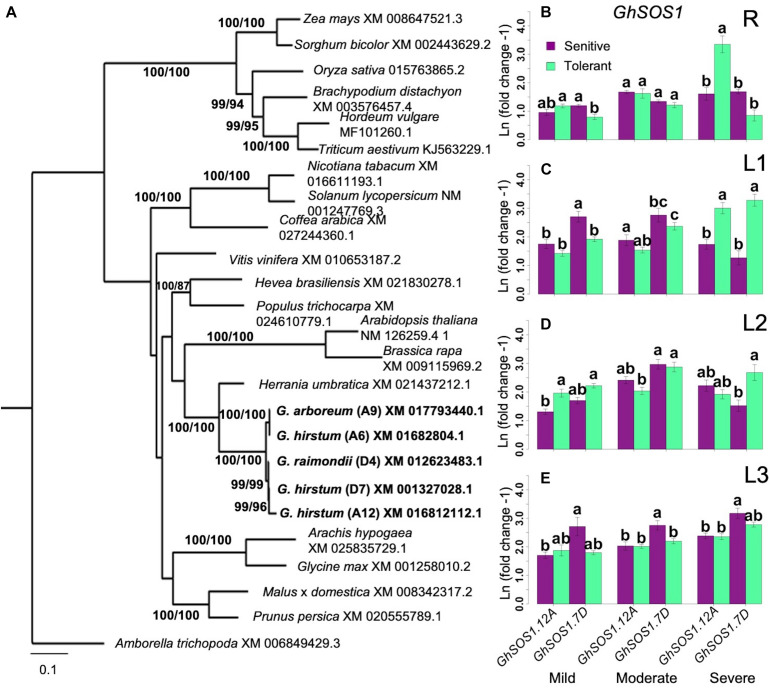
Analysis of *SOS1* homology and expression. **(A)**, Identification of orthologous *GhSOS1.* Bootstrap values are ML/MP. CI = 0.475; RI = 0.5899. **(B–E)**, Spatial expression profiles of orthologous *GhSOS1* genes in sensitive and tolerant genotypes along the vertical axis of the plant (**B** = roots/R; **C** = leaves/L1; **D** = leaves/L2; **E** = shoot/L3). Transcript abundance stayed relatively consistent throughout the stress period along the vertical axis of the plant. The A-subgenome ortholog on chromosome-6 was expressed at half the level in the orthologs on chromosomes-12 and chromosome-20. Comparable expression of *GhSOS1.A12* and *GhSOS1.D20* initially suggested no expression bias between subgenomic orthologs. According to phylogeny, the *GhSOS1.A12* is most closely related to a gene from *G. raimondii.* Regardless, expression profiles were consistent between tolerant and sensitive genotypes (Groups assigned by Tukey pairwise comparisons). Error bars indicate standard error.

*SOS1*, which encodes a plasma membrane Na^+^/H^+^ antiporter, facilitates Na^+^ efflux and a critical component of SOS-signaling pathway for regulating cellular Na^+^ homeostasis (Apse and Blumwald, 2007; Ji et al., 2013). BLAST queries identified three orthologs of the Arabidopsis *AtSOS1* on chromosome-6 (A6 in subgenome-A; *GhSOS1.A6*), chromosome-12 (A12 in subgenome-A; *GhSOS1.A12*), and chromosome-20 (D7 on subgenome-D; *GhSOS1.D7*) of *G. hirsutum* (Figure 6A). While *GhSOS1.A6* formed a clade with 100% bootstrap support with *G. arboreum*, the *GhSOS1.A12* and *GhSOS1.D7* formed a clade with 99% bootstrap support with *G. raimondii.*

In the roots (R), expression of all *GhSOS1* orthologs was not affected by mild salt stress in both inferior and superior genotypes. Slight upregulation of *GhSOS1.A12* and *GhSOS1.D7* were detectable at moderate salt stress (Figure 6B). Under severe stress, *GhSOS1.A12* exhibited a unique pattern of expression relative to the two other *GhSOS1* orthologs, with significant upregulation specific to the superior genotypes. This positive correlation suggests genotype-specific mode of regulation of *GhSOS1.A12*, and that this gene may have more important contribution to the differential profiles of Na^+^ accumulation in the roots of inferior and superior cultivars, perhaps as a function of the balance between uptake and efflux (Figure 4A).

Complex patterns of *GhSOS1* expression were observed along the L1-L2-L3 axis. Upregulation was detectable for *GhSOS1.A12* and *GhSOS1.D7* under moderate stress, with a tendency for slightly higher magnitude of upregulation in superior genotypes. Interestingly, under severe stress, expression of *GhSOS1.A12* and *GhSOS1.D7* tapered off in older L1 leaves of inferior genotypes but not in superior genotypes where transcript levels continued to rise relative to the levels under moderate stress (Figure 6C). In younger leaves (L2, L3), different magnitudes of upregulation of both *GhSOS1.A12* and *GhSOS1.D7* were still evident under moderate to severe stress in both inferior and superior genotypes (Figures 6D,E). While the temporal and spatial patterns of Na^+^ accumulation in the vertical shoot axis (Figure 4B) indicated higher levels of accumulation in superior than inferior genotypes across all stress levels, the trends in *GhSOS1.A12* and *GhSOS1.D7* expression tend to suggest that these genes may be involved in some mechanisms that ameliorate the cellular toxicity of excess cytoplasmic Na^+^, perhaps through efflux across the plasma membrane. It also appears that this mechanism is equally functional in both inferior and superior genotypes, albeit at different magnitudes. We did not detect any expression of *GhSOS1.A6* in the roots or the shoots of the control or under stress conditions.

### Differential Expression of *NHX1* Genes

**FIGURE 7:**
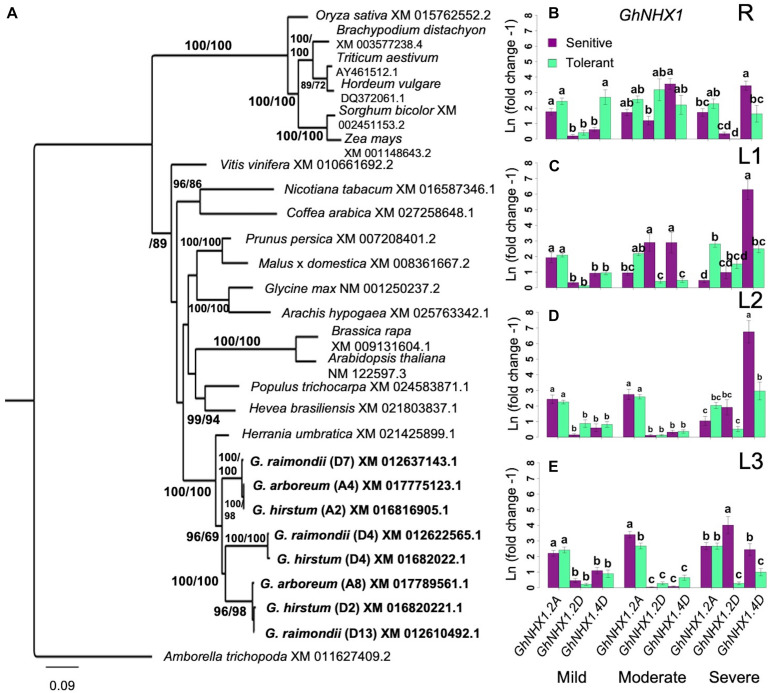
Analysis of *NHX1* homology and expression. **(A)**, Identification of orthologous *GhNHX1.* Bootstrap values are ML/MP. CI = 0.4592; RI = 0.6256. **(B–E)**, Spatial expression profiles of orthologous *GhSOS1* genes in sensitive and tolerant genotypes along the vertical axis of the plant (**B** = roots/R; **C** = leaves/L1; **D** = leaves/L2; **E** = leaves/L3). Increases in gene expression was sporadic without overall patterns related to possible variances associated with position along the vertical axis of the plant, stress levels, or tolerance category based on SES. Under severe stress in L1, there the D-subgenome ortholog was upregulated in the sensitive genotypes. There was also a consistent upregulation of the A-subgenome ortholog in L3 in the tolerant genotype (Groups assigned by Tukey pairwise comparisons). Bootstrap values are ML/MP. Error bars indicate standard error. Error bars indicate standard error.

Sequestration of excessive Na^+^ into the vacuolar compartments through the Na^+^/H^+^ class of endosomal antiporters encoded by the *NHX* gene family has been shown to increase salt tolerance in Arabidopsis through Na^+^/H^+^ and K^+^/H^+^ exchange (Apse et al., 1999). BLAST queries identified three potential orthologs of the Arabidopsis *AtNHX1* in *G. hirsutum*, located on chromosome-2 (A2 in subgenome-A; *GhNHX1.A2*), chromosome-15 (D2 in subgenome-D; *GhNHX1.D2*), and chromosome-17 (D4 in subgenome-D; *GhNHX1.D4*) (Figure 7A). The *GhNHX1.A2* and *GhNHX1.D2* formed clades with 100% bootstrap support with both ancestral *G. arboreum* and *G. raimondii*. *GhNHX1.D4* only formed a clade with *G. raimondii.*

Compared to *GhHKT1* and *GhSOS1*, the profiles of *GhNHX1* showed greater spatio-temporal variation between inferior and superior genotypes across stress levels. In the roots (R), upregulation of *GhNHX1.A2* in both the tolerant and sensitive genotypes and *GhNHX1.D4* only in superior genotypes were detected under mild stress (Figure 7B). However, increase in expression became more evident for one or more *GhNHX1* orthologs in both inferior and superior genotypes under moderate and severe levels of stress. These trends indicate that while Na^+^ content in the roots vary significantly under all stress levels between inferior and superior genotypes, Na^+^ sequestration to vacuolar compartments appeared to be equally functional in both inferior and superior genotypes. Different *GhNHX1* orthologs also seem to be under distinct regulatory controls in different genetic backgrounds and may be providing some complementation effects.

The expression profiles of *GhNHX1* genes was consistent throughout the shoots at mild stress (Figures 7C–E). This pattern continued into moderate stress in the younger shoots: L2 and L3. In the oldest leaves (L1), *GhHNX1.2D* and *GhHNX1.4D* were upregulated in the sensitive shoots. At severe stress in the older shoots (L1 and L2), *GhHNX1.4D* had the dominate expression profile in the sensitive shoots. In the youngest shoots (L3), *GhNHX1* expression was still predominate in the sensitive shoots but spread out across the three *GhNHX1* genes. Similar to the trends observed in the roots, it appears that different *GhNHX1* orthologs have different patterns of regulation, and the functions of these orthologs may be complementary across different genotypes. The general trends revealed from the spatio-temporal patterns of *GhNHX1* across inferior and superior genotypes suggest that the mechanism of vacuolar Na^+^ sequestration by NHX1-type pumps are equally functional in both inferior and superior genotypes, hence could not fully explain the observed differential Na^+^ accumulation in the vertical shoot axis of the plant relative to the magnitude of stress sensitivity.

### Na^+^ and K^+^ Recirculation Capacity of Sensitive and Tolerant Genotypes

**FIGURE 8:**
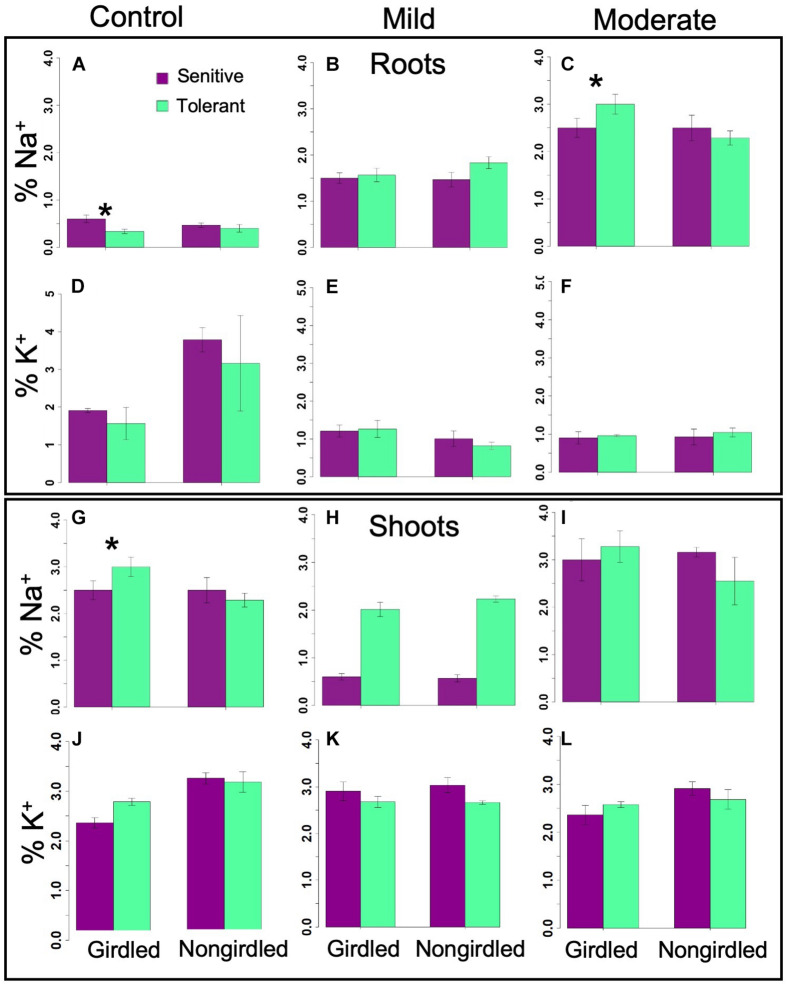
Bar graphs showing the Na^+^ and K^+^ content in the phloem of girdled and non-girdled plants under mild and moderate stresses in the roots **(A–F)** and shoots **(G–L)**. Insignificant differences in ion content suggested that recirculation is not critical to maximizing the tolerance potential (Table 2). Error bars indicate standard error (*significant at *p* < 0.05).

In a non-uniform root zone experiment, it was previously shown that Na^+^ taken-up by a set of roots in a high-concentration salt solution could be transported by the phloem to a second set of roots connected to the same shoot in a low-concentration salt solution (Kong et al., 2012). This transport was blocked by girdling the plants that severs the phloem. The hypothesis that tolerant genotypes are more efficient at Na^+^ recirculation from the shoots back to the roots was tested by having the inferior and superior genotypes girdled under control and stress (*i.e.*, EC∼20dS/m to EC∼40dS/m) conditions. If the tolerant genotypes were more efficient at Na^+^ recirculation, the shoots of its non-girdled plant under stress would have proportionally less Na^+^ relative to its girdled counterpart, and also relative to the sensitive non-girdled and girdled genotypes. Although there weresignificant differences in Na^+^ concentration between inferior and superior genotypes at different stress levels, differences in the shoot Na^+^ or K^+^ content between girdled and non-girdled plants were not statistically significant (Figure 8 and Table 2). In general, the girdled plant declined faster than the non-girdled plants and were not as productive under control conditions. However, this decline could not be attributed to differences in Na^+^ uptake or removal from the shoots. Overall, the results of the girdling experiment implied that superior genotypes may be more efficient in the uptake of Na^+^, but more efficient Na^+^ elimination is not a critical factor in stress avoidance. These results are consistent with the non-significant differences in the expression of *GhHKT1, GhSOS1*, and *GhNHX1* (Figures 5–7).

**TABLE 2:** Analysis of variance table for Na^+^ accumulation in according to genotype, girdling effect, and salinity level as well as their interacting terms.

Source	Df	Sum Sq.	Mean Sq.	*F*-Value	*p*
Genotype	1	2.74	2.74	13.9762	0.0005311
Girdling	1	0.087	0.087	0.4447	0.5083594
Stress Level	2	64.908	32.454	165.5705	< 2.2E-16
Genotype * Girdling	1	0.617	0.617	3.15	0.0828465
Genotype * Stress Level	2	8.115	4.058	20.7012	4.61E-07
Girdling * Stress Level	2	0.294	0.147	0.7497	0.4784364
Genotype * Girdling * Stress Level	2	0.907	0.454	2.3143	0.1107483
Residuals	44	8.625	0.196		

*Both genotype and stress levels show significant differences in Na^+^ accumulation and their interactions are significant. Girdling effect did not contribute to significant differences in Na^+^ accumulation nor were there any significant interactions with the other stress parameters.*

### Salt Tolerance Potential Explained by Multi-Factor Interaction

The *randomForest* analysis was used to integrate all physiological, biochemical and molecular properties across the minimal comparative panel in order to reveal patterns that mimicked the general trends uncovered by the principal components (Figure 3 and Table 3). Among the attributes measured along the L1-L2-L3 axis, variation in total tissue Mg^2+^ content, dry weight, CAT, PER, stomatal conductance, *GhNHX1* expression, total proline content and chlorophyll content had the most significant contributions to the total phenotypic variance. The contributions of tissue Mg^2+^ content, dry weight and stomatal conductance to the phenotypic variance remained relatively constant as stress increased in severity. The importance of maintaining chlorophyll stability, antioxidant capacity, membrane lipid protection, proline content and *GhNHX1*/*GhSOS1* expression tended to increase with increasing severity of stress, while CAT and PER explained the variance more during milder stress and less during severe stress. Among the various roots (R) parameters used to assess the dominant contributors to total phenotypic variance, PER, Na^+^ content, dry weight, total proline content, lipid peroxidation and *GhHKT1* expression appeared to be the most important as their profiles were generally less affected by the severity of stress. The relative importance of CAT and PER as well as antioxidant capacity appeared to decline with increasing severity of stress hence contributed less to phenotypic variance.

**TABLE 3:** Importance of variables as determined by random forest regression tree modeling.

Variable	All Shoots	Shoots Mild	Shoots Moderate	Shoots Severe	All Roots	Roots Mild	Roots Moderate	Roots Severe
Potassium	3.786691	1.2357299	0.7314199	1.413495	1.767395	0.6950132	**1.2141868**	0.4615078
Magnesium	**10.074909**	**1.5416042**	**3.7099761**	**2.149361**	1.104456	0.3501201	0.4394079	0.4401129
Calcium	4.333448	0.6993617	**1.5302499**	1.020173	1.651431	**0.9367376**	0.5013073	0.7429788
Sodium	3.996921	0.9466907	0.8603565	1.433828	**3.200775**	0.6087103	0.6511365	0.7933997
Chlorophyll	**5.407807**	0.9928562	**1.5064567**	**1.631328**	N/A	N/A	N/A	N/A
Conductance	**6.428687**	**2.7319109**	**2.7380638**	**1.581265**	N/A	N/A	N/A	N/A
ELI	4.158879	**2.1764977**	0.94189	1.376287	N/A	N/A	N/A	N/A
Catalase	**6.903182**	**1.405166**	**3.1741731**	1.19035	1.736685	0.6711793	0.6007781	**0.9912815**
Total Antioxidants	4.533538	0.9706393	0.9726165	**1.711684**	2.0168	0.4534602	0.3882435	**0.9208404**
Lipid Peroxidation	4.790778	0.7779982	**1.7108021**	**1.731511**	**2.557239**	0.5387114	0.4153387	0.6176625
Peroxide	3.44201	**1.6375462**	1.1355657	1.188315	1.936919	0.6224218	**1.1031374**	0.4271131
Peroxidase	**6.434328**	**4.2004274**	1.0585364	**2.60553**	**3.434178**	0.648062	0.7395362	**0.8612855**
Proline	**6.036576**	**2.0171469**	1.3707109	**4.153838**	**2.728543**	0.688928	0.6728211	0.4921828
Dry Weight	**8.642007**	**5.5706243**	**6.2804716**	**1.905753**	**2.76899**	**1.4086769**	0.8048128	0.3612618
HKT1	3.565912	1.229558	1.0126279	1.041146	**2.119004**	0.5296454	0.6652772	0.7215812
NHX1	**6.147264**	**1.4360769**	1.0945722	**2.175037**	1.865067	0.5421	0.5770708	0.6909742
SOS1	4.518785	1.0602486	0.8654274	**2.399682**	1.829184	0.6002338	0.5819456	0.7568177

*In bold are the parameters that contribute the most toward stress tolerance for the combined roots and shoots, as well as, for each time-point and level of salinity (Mild salinity, EC∼20dS/m; Moderate salinity, EC∼40dS/m; Severe salinity, EC∼58dS/m).*

**FIGURE 9:**
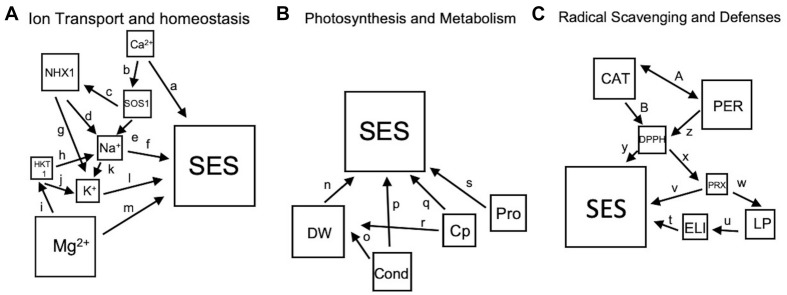
**(A–C)**, Theoretical models based on referenced studies listed in Supplementary Table 2 and empirical data from this study. The sizes of the squares are in accordance with their overall importance for shoot data as determined by *randomForest* analysis (Table 3). Single-ended arrows are causal relationships, and double-sided arrows indicate relationships that co-vary. The abbreviations are listed in the Materials and Methods.

Using the theoretical models as input for path analysis (Figures 9A–C), the importance of both shoot Mg^2+^ and Ca^2+^ contents were emphasized. Based on individual coefficients (Table 4), Mg^2+^ content correlated positively with SES at all stages of stress, while Ca^2+^ content negatively correlated with tolerance. At medium stress level (EC∼40dS/m), Ca^2+^ being an activator of the SOS pathway, had a positive correlation with *GhSOS1* expression, which in turn had a positive correlation with *GhNHX1* expression and Na^+^ content. These trends suggest that the SOS1 pathway was more efficient in promoting tolerance at moderate stress levels. At the most severe stress levels, *GhSOS1* expression negatively correlated with *GhNHX1* expression, perhaps because the pathway has already been established, and both *GhSOS1* expression and *GhNHX1* expression negatively correlated with Na^+^ content. Na^+^ content at severe stress was significantly correlated with SES (Figure 4D).

**TABLE 4:** Path analysis paths and regressions coefficients.

Path	Shoots			Roots

	Mild	Moderate	Severe	All
	**Ion Transport and Homeostasis**			
Ca^2+^ - > SES (a)	**−7.998**	**−4.830**	**−5.981**	**−2.228**
Ca^2+^ - > SOS1 (b)	**−**1.557	**2.760**	**−**0.718	0.848
SOS1 - > NHX1 (c)	**−**1.549	**5.520**	**−2.915**	0.126
NHX1 - > Na^+^ (d)	1.149	**−**0.714	**−3.226**	**−2.436**
SOS1 - > Na^+^ (e)	**−**0.527	**2.366**	**−3.050**	**3.214**
Na^+^ - > SES (f)	**−**1.121	0.521	**2.527**	**−6.911**
NHX1 - > K^+^ (g)	**−2.484**	**−**0.622	0.639	**−3.728**
HKT1 - > Na^+^ (h)	**−**1.759	**−**1.402	**−**1.537	**−2.712**
Mg^2+^ - > HKT1 (i)	0.393	**−**0.762	0.675	**−**1.36
HKT1 - > K^+^ (j)	1.738	0.803	0.965	**−**0.735
Na^+^ - > K^+^ (k)	**−4.169**	1.862	0.224	**−2.454**
K^+^ - > SES (l)	**−2.300**	**−**0.815	**−**0.886	**−4.519**
Mg^2+^ - > SES (m)	**12.946**	**8.206**	**8.907**	0.038

	**Photosynthesis and Metabolism**			

DW - > SES (n)	**−2.967**	1.205	**9.791**	**4.602**
Cond - > DW (o)	**3.578**	**−2.046**	**−**0.237	**−**0.242
Cond - > SES (p)	**6.188**	**5.242**	**3.078**	**−**0.188
Cp - > SES (q)	1.775	**2.803**	**5.22**	0.785
Cp - > DW (r)	**1.991**	**−**0.883	1.827	1.914
Pro - > SES (s)	**2.906**	**−**0.399	**2.162**	**3.372**

	**Radical Scavenging and Defenses**			

ELI - > SES (t)	**2.067**	**−**0.871	1.288	N/A
LP - > ELI (u)	**−2.699**	0.56	**−**0.622	**−**0.506
PRX - > SES (v)	**−**0.919	**−2.407**	**−2.531**	**−3.772**
PRX - > LP (w)	0.219	1.17	0.32	**−2.795**
DPPH - > PRX (x)	**−**1.265	**−**0.569	**−3.307**	**−**0.389
DPPH - > SES (y)	0.467	**4.351**	**3.276**	**4.132**
PER - > DPPH (z)	**−**0.147	**−**1.332	**−**0.904	**4.346**
CAT < - > PER (A)	**−**0.077	**−2.59**	**−3.409**	**−**1.108
CAT - > DPPH (B)	**−**0.263	0.414	**−**1.153	**−**1.064

*Regression coefficients given as *z*-scores. *Z*-scores ≥ 1.96 or ≤ **−**1.96 are significant at *P* ≤ 0.05. Significant *z*-scores are also in bold. (Mild salinity, EC∼20dS/m; Moderate salinity, EC∼40dS/m; Severe salinity, EC∼58dS/m).*

In the roots, both Na^+^ and K^+^ contents are strongly negatively correlated with SES (Figure 4E). Gene expression in general negatively correlated with Na^+^ and K^+^ concentrations. However, expression of *GhSOS1* increased with increasing Na^+^ concentration. Interestingly, Mg^2+^ and Ca^2+^ do not appear to be significant factors to tolerance in the roots. The overall trend, with the exception of moderate stress in the shoots, ion transporters have minimal impact on differential stress tolerance across genotypes. All the models for ion transport and homeostasis had non-significant goodness of fit indices indicating a poor fit to the proposed model (Table 5). To improve the model, unmeasured parameters such as the transporter genes that preferentially select Mg^2+^ over Ca^2+^ could be included and the non-significant parameters removed. The models do however explain 39% to 62% of the total variance in SES driven mostly by Mg^2+^ and Ca^2+^ in the shoots, and 32% driven mostly by negative interactions in the roots.

**TABLE 5:** Path analysis goodness of fit measures.

		Shoots		Roots

Fit Tests	Mild	Moderate	Severe	All
**Ion and Ion Transport**				
Chi-square (df)	(15) 242.248	(15) 1843.894	(15) 218.42	(15) 83.206
RMSEA	0.324% CI [0.289,0.361]	0.280% CI [0.244,0.317]	0.304% CI [0.272,0.344]	0.178 90% CI[0.142,0.216]
SRMR	0.28	0.194	0.209	0.133
CFI	0.265	0.286	0.227	0.511
TLI	**−**0.372	**−**0.333	**−**0.443	0.087
NFI	0.282	0.305	0.250	0.4503
SES	***R*^2^ = 0.622**	***R*^2^ = 0.388**	***R*^2^ = 0.459**	***R*^2^ = 0.316**
K^+^	***R*^2^ = 0.180**	*R*^2^ = 0.028	*R*^2^ = 0.008	***R*^2^ = 0.106**
Na^+^	*R*^2^ = 0.033	*R*^2^ = 0.050	***R*^2^ = 0.113**	***R*^2^ = 0.140**

**Photosynthesis and Metabolism**				

Chi-square (df)	**(4) 2.772**	**(4) 9.181**	(4) 9.569	(4) 32.623
RMSEA	**0.000 90% CI [0.000,0.106]**	**0.095 90% CI [0.000,0.177]**	0.098 90% CI [0.008,0.180]	0.223 90% CI [0.156,0.297]
SRMR	**0.041**	**0.064**	**0.067**	0.121
CFI	**1**	0.855	**0.949**	0.493
TLI	**1.063**	0.638	0.872	-0.268
NFI	**0.953**	0.799	0.919	0.509
SES	***R*^2^ = 0.258**	***R*^2^ = 0.196**	***R*^2^ = 0.518**	***R*^2^ = 0.388**
Dry Weight	***R*^2^ = 0.104**	*R*^2^ = 0.033	*R*^2^ = 0.023	*R*^2^ = 0.039

**ROS and Antioxidants**				

Chi-square (df)	(12) 68.366	(12) 65.640	(12) 72.175	(7) 32.850
RMSEA	0.181 90% CI [0.140,0.223]	0.176 90% CI [0.136,0.219]	0.187 90% CI [0.146,0.229]	0.160 90% CI [0.107,0.217]
SRMR	0.114	0.128	0.13	0.095
CFI	0.078	0.344	0.396	0.653
TLI	**−**0.613	**−**0.148	**−**0.058	0.257
NFI	0.168	0.361	0.401	0.633
SES	*R*^2^ = 0.036	***R*^2^ = 0.156**	***R*^2^ = 0.147**	***R*^2^ = 0.184**
Peroxide	*R*^2^ = 0.011	*R*^2^ = 0.002	*R*^2^ = 0.071	*R*^2^ = 0.001
DPPH	*R*^2^ = 0.001	*R*^2^ = 0.016	*R*^2^ = 0.012	***R*^2^ = 0.128**

*Root Mean Square Error of Approximation (RMSEA) is significant at ≥ 0.90. Standardized Root Mean Square Residual (SRMS) is significant at < 0.08. Comparative Fit Index (CFI) ≥ 0.90. Tucker Lewis Index (TLI) ≥ 0.95. Normed-Fit Index (NFI) ≥ 0.95. Significant fit indices are bold.*

The models for photosynthesis and metabolism in shoots have at least two fit indices that are significant (Table 5). Stomatal conductance is a clear indicator of stress tolerance at all stress levels. Chlorophyll content is also a good indicator but mostly later during senescence at higher stress levels. Dry weight at the beginning of the experiment was negatively correlated with SES but non-significant at moderate stress and strongly positively correlated at severe stress. This would substantiate the observation that the most vigorous genotypes under control conditions were also the most sensitive to stress, which further suggests that the underlying growth regulatory mechanisms may be adventitious under stress conditions. Proline content in the shoots had an unusual pattern of being important early in stress, non-significant at moderate stress, and significant under severe stress. Overall, the models explained 25.8, 19.6, and 51.8% of the variance in SES at mild, moderate, and severe stress, respectively. The root model for photosynthesis and metabolism had no significant fit indices, but the dry weight and proline content were strongly correlated with SES, explaining 38.8% of the variance for SES.

None of the models for radical scavenging and defense had significant fit indices but suggested the importance of antioxidants at moderate and severe stresses. At moderate stress, total antioxidants were strongly positively correlated with SES. At the same time, PRX was negatively correlated with SES. These trends continued through severe stress when total antioxidants had a negative correlation with PRX. In the shoots, neither PER nor CAT had a significant impact on antioxidant content. This suggests that other radical scavenging mechanisms could be operating in tolerant genotypes. The roots had similar correlations of antioxidant and PRX contents, except that the antioxidant content was mostly driven by PER. Overall, the models explained less of the variation in SES, with 15.6, 14.7, and 18.4% in moderate shoots, severe shoots, and roots, respectively.

## Discussion

As the understanding of the fundamental mechanisms of salinity tolerance continued to advance through functional and comparative genomics in both model and crop plants, the paradigm of a single or few genes controlling the complex physiological and biochemical bases of adaptive responses has gradually shifted to system-wide or holistic paradigm. The modern views suggest that stress tolerance potential is the manifestation of complex interacting biochemical pathways and genetic networks that work differentially or in concert to generate varied responses to stress conditions, hence no consensus combinations or subsets of parameters could fully explain phenotypic variation within and across species as each mechanism tends to be unique (Wang et al., 2017; Morton et al., 2019). This view is also consistent with the new paradigms of the *Omnigenic Theory* for quantitative traits, which proposed the additive and synergistic effects of several core genes and hundreds if not thousands of peripheral or trans-effect genes (Boyle et al., 2017). Such level of complexity was apparent from the differential responses observed across the cotton germplasm, a plant species known for its inherently high baseline level of tolerance to salinity and drought. This was evident from the high input NaCl concentrations necessary to elicit differential responses at the whole-plant level across a meaningful genetic diversity panel.

### Conservation of the First-Line-of-Defense

While the milder salinity treatments (EC∼20dS/m) during the optimization experiment did not clearly distinguish the sensitive and tolerant genotypes, distinct biochemical and physiological transformations apparently have taken place for either short-term or long-term adaptive mechanisms. For instance, while the total tissue PRX begins to build-up in the sensitive genotypes, potentially causing damage to membranes, the tolerant genotypes respond by producing more antioxidants, efficient stomatal conductance, and milder losses in chlorophyll content hence more robust photosynthesis. These responses to salinity stress have been observed in other salt-tolerant genotypes of cotton (Wang et al., 2017) as well as in other plant species (Chakraborty et al., 2016). These trends also showed correlations with reduced tissue Na^+^ and increased K^+^ when compared to more sensitive genotypes. Interestingly, results of this study also showed salinity-induced increase in Mg^2+^ in the tissues of tolerant genotypes, which was also observed previously among halophytes subjected to NaCl concentrations above 400 mM (Vicente et al., 2004). Mg^2+^ is an activator of HKT-type transporters including HKT1 during the facilitation of Na^+^ influx to the roots and recirculation in the phloem (Chen et al., 2017). Results of this study showed that *GhHKT1* expression did not increase with increased Mg^2+^ level (Table 4), which is an important component of initial signaling through a cascade of protein phosphorylation. Mg^2+^ maybe involved in defense pathways activated at moderate and severe stress levels, a role that is often attributed to Ca^2+^. Results of this study clearly indicated that Ca^2+^ was negatively associated with tolerance and appeared to be important only at moderate salinity (*i.e.*, EC∼40dS/m) (Chinnusamy et al., 2004). While our empirical models did not compare Mg^2+^ and chlorophyll contents directly, the more tolerant genotypes were able to maintain higher levels of chlorophyll and delay senescence under severe salinity. While the total Mg^2+^ content in control plants was consistent across salinity tolerance categories, there was only a slight increase in tolerant plants under stress. Much of the differences in Mg^2+^ content was due to significant losses in the more sensitive plants, which may have corresponded to a loss of chlorophyll. This is another strong indication of the ability of the tolerant genotypes to maintain close to normal cellular homeostasis under levels of salinity that are already deleterious to sensitive genotypes.

An alternative hypothesis that could address the importance of Mg^2+^ is the possibility that it may be important largely for osmotic adjustment and reduction of ionic toxicity. Efficient means for maintaining optimal osmotic balance has been implicated with changes in the levels of compatible osmolytes such as proline. While this may appear to be beneficial to the plant as part of the first-line-of-defense or short-term defense, production of these osmolytes often comes with major metabolic expense (Raorane et al., 2015; Theerawitaya et al., 2015). Our current data suggest that increased production of proline during the early stages of stress may cause some trade-offs. Nevertheless, plants that tolerate much higher levels of salinity still had more proline in both the roots and shoots.

Despite the established knowledge, the general trends revealed in the current study implied that increased ionic concentrations do not necessarily translate to greater expression of the major facilitators of Na^+^ homeostasis such as *GhHKT1*, *GhSOS1* and *GhNHX1* in the more tolerant cultivars at moderate to severe levels of salinity. With the exception of *GhHKT1* and *GhSOS1* expression in the roots, there was no clear evidence especially in the shoots that *GhHKT1*, *GhSOS1* and *GhNHX1* expression were critical to the observed phenotypic variances. Increased expression of *GhNHX1* in the shoots at severe salinity (EC∼58dS/m) was coincident with lower Na^+^ concentration when compared with the tolerant genotypes with lower *GhNHX1* expression profiles. We also observed a significant reduction in the concentration of K^+^ in the roots at mild salinity (EC∼20dS/m) (Table 4; Bassil et al., 2011). At moderate salinity (EC∼40dS/m), *GhSOS1* expression increased with Na^+^ concentration, but eventually declined with continued stress progression to EC∼58dS/m. *GhHKT1* expression had no correlation with either Na^+^ or K^+^ content. It has been previously observed that peak expression of *GhHKT1*, *GhSOS1* and *GhNHX1* occurred at 5–20 h after the onset of stress, the expression levels went back to normal or below control levels by 24 h (Wang et al., 2017). The GhHKT1, GhSOS1 and GhNHX1 proteins may occur early during stress and contribute to the baseline tolerance, hence further production may not be necessary even if the stress continue to intensify.

We have also observed that even the most tolerant genotypes were not necessarily immune to Na^+^ toxicity. The *first-line-of-defense* (*i.e.*, sequestration mechanisms) may delay the onset of injuries, but irreversible injuries occur at a much later period during chronic exposure. In the principal component analysis of various physio-morphometric attributes, the profiles of tolerant genotypes under severe salinity tended to overlap with the profiles of sensitive genotypes under milder salinity. This implied that sensitive and tolerant genotypes have similar biochemical and physiological responses occurring at different thresholds of osmotic imbalance or ionic toxicity. However, a separate plot for the profiles under severe salinity showed that the traits that are much more closely associated to tolerance such as proline content were still more dominant among tolerant genotypes. Interestingly, while one of the principal components (PC2) segregated the sensitive and tolerant genotypes, separation was much clearer based on the spatial similarities and differences in the L1-L2-L3 axis of the plant (Figure 3C). As senescence begins in the oldest L1 leaves representing the onset of chlorophyll degradation, such pattern also corresponded to the profile of Mg^2+^ accumulation in tolerant genotypes. At severe salinity, there is a clear vertical partitioning of resources, consistent with what has been observed in previous studies in maize (AbdElgawad et al., 2016).

### Cellular Na^+^ Homeostasis Represents Only the Baseline of the Untapped Potentials Across the Cultivated Germplasm

The inherent capacity for selective uptake of K^+^ over Na^+^ (*HKT1*), Na^+^ exclusion (*SOS1*), and/or avoidance by subcellular sequestration (*NHX1*) have been viewed among the major contributors to the variances for adaptive responses across a gradient of tolerance/sensitivity. It is also generally accepted that uptake of Na^+^ by the roots occurs either through passive transport facilitated by non-selective cation transporters, through K^+^ transporters that do not discriminate against Na^+^ when its concentration is high, or through a combination of both mechanisms (Apse and Blumwald, 2007; Davenport et al., 2007; Kronzucker and Britto, 2011). Although there are mechanisms that allow some of the absorbed Na^+^ to be extruded back externally via Na^+^/H^+^ antiporters like SOS1, it has been hypothesized that different plant species and genotypes have inherently unique capacities for controlling the balance between uptake, extrusion, xylem unloading, and intercellular and intracellular mobilization (Wang et al., 2017). This balance is presumed to be behind the variation in the net Na^+^ accumulation in sensitive organs.

Mechanisms for ameliorating the negative impacts of ionic toxicity and osmotic imbalance at the cellular level are based on functional genomic studies in the model Arabidopsis. These studies highlighted the central role of avoidance by maintaining tolerable levels of Na^+^ in the cytoplasm. It has been shown that the more tolerant genotypes tend to accumulate less Na^+^ than sensitive genotypes (Horie et al., 2009; Munns et al., 2012), or that ionic toxicity is mitigated when excess Na^+^ is sequestered in the vacuolar compartment (Apse et al., 1999; Wu et al., 2004). It was also proposed that the mechanisms for differential accumulation of Na^+^ could be spatially controlled by retaining much of the Na^+^ absorbed through the roots to the least fragile and relatively more dispensable sink organs along the vertical shoot axis such as the oldest leaves. This capacity is expected to occur in more tolerant genotypes (Cheeseman, 1988; Munns and Tester, 2008). Conversely, there are claims that the more tolerant genotypes have a general tendency to accumulate more ions under stress, contending that increased ion concentration is necessary to maintain osmotic balance, thereby facilitating new growth. Specifically highlighted in those studies was the maintenance of lower Na^+^/K^+^ ratio or higher K^+^/Na^+^ (Chen et al., 2007; Shabala et al., 2010). The vertical movements of Na^+^ and K^+^ have been associated with the HKT1-class of ion transporters, which function for ion removal or loading from the xylem or into the phloem for recirculation back to roots and eventual extrusion.

The spatio-temporal profiles showed that tolerant genotypes did accumulate more Na^+^ in the shoots compared to sensitive genotypes. This difference was most apparent in the more labile upper/younger organs of the shoot vertical axis (L2-L3) especially at high levels of salinity. However, accumulation of Na^+^ in the shoots did not vary significantly in the L1-L2-L3 profile, and other than magnitude, there was no significant difference in vertical distribution associated with tolerance (Figures 4B,C). This was consistent with the general trends in *GhHKT1* expression in shoot/leaves that showed increased expression particularly under severe salinity (Figures 5C–E). As expected, *GhHKT1* expression was highest in L1, but this level of expression did not translate to significant increase in Na^+^ accumulation. There was also no significant difference in *GhHKT1* expression in the shoot/leaves at any stage of stress between the sensitive and tolerant genotypes.

While there was an increase in shoot Na^+^ concentrations, such increases were significantly lower in the roots of tolerant genotypes compared to sensitive genotypes, and the expression of *GhHKT1.A1* was significantly upregulated in tolerant genotypes. If Na^+^ was being withdrawn from the xylem sap at a higher rate in tolerant genotypes, then Na^+^ in the roots should increase at a higher rate. Na^+^ in the shoot/leaves should also increase at a lower rate. Since *GhHKT1.A1* is co-upregulated with *GhSOS1.A12* in the tolerant genotype under severe salinity, it is possible that Na^+^ in the roots was being eliminated from the plant. Had this occurred though, there would have been a reduction in the rate of Na^+^ accumulation in the shoots. It has also been hypothesized that SOS1 in the roots functions by loading Na^+^ into the xylem (Zhang et al., 2017; Wang et al., 2020). Considering the converse activity of *GhHKT1.A1*, the relative expression of *GhSOS1.A12* was slightly higher in the roots of the tolerant genotypes under severe stress and may have resulted in both reduced Na^+^ in the root and increased Na^+^ in the shoot.

K^+^ concentrations remained constant along the L1-L2-L3 axis but declined exponentially in the roots (Figures 4E–H). The magnitude of such decline at higher levels of salinity was more pronounced in sensitive genotypes. Because shoot K^+^ remained constant, the Na^+^/K^+^ ratio increased with Na^+^ concentrations. The increasing Na^+^/K^+^ was more pronounced in the roots due to the decreasing K^+^ concentrations, coupled with the increase in Na^+^ and being immersed in an environment of ever-increasing Na^+^/K^+^ ratio in the input solution, *i.e.*, 45/1 at mild salinity, EC = 20dS/m, 90/1 at EC = 40dS/m moderate salinity, and 135/1 at severe salinity, nearly 10-times higher than the ratios in the sensitive genotypes. Assuming that shoot K^+^ concentrations were stable, the activity of *GhHKT1.A1* in the roots would have been unrelated to K^+^ concentration since it did not change through the course of the experiment. However, if K^+^ leaching did occur in association with transpiration, then *GhHKT1.A1* activity would be necessary to maintain a constant shoot K^+^ concentration and may account for the reduction in the roots.

The downregulation of *GhHKT1* in the shoots under mild to moderate salinity suggests that Na^+^ recirculation to the roots is not a significant contributor to the variation across the comparative panel. This was confirmed by the phloem girdling experiment, which showed no significant differences in Na^+^ accumulation between girdled and non-girdled plants (Figure 8). The activity of *GhHKT1* however did increase in the shoots at severe salinity, and thus Na^+^ recirculation may occur universally late during the progression of stress. Neither the gene expression data nor Na^+^ content data would suggest this has a differential contribution to the observed phenotypic variation. We were unable to continue the girdled plant experiment into severe stress levels because the girdled plant incurred more stress injuries than the non-girdled plants. One effect of girdling was a plant with a severed phloem did not react instantaneously to the osmotic shock at the onset of stress. This was typical of all cultivars with intact phloem, where wilting was apparent within minutes of stress application.

When the total Na^+^ accumulation in roots and shoot/leaves were combined, there were little difference between sensitive and tolerant genotypes, implying that the capacity for exclusion is consistent across the minimal comparative panel. As mentioned above, Na^+^ accumulation was distributed differentially in sensitive compared to tolerant genotypes, with more Na^+^ in the roots and less in the shoots and accompanied by an increase in *GhSOS1.A12* expression in the roots. *GhSOS1.A12* and *GhSOS1.D7* were also upregulated in the lower L1 leaves under severe salinity in tolerant genotypes. Otherwise, in the upper portions of the shoot axis (L2-L3), there was no significant difference in *GhSOS1* expression (Figures 6D,E). Differences in *GhSOS1.A12* and *GhSOS1.D7* expression may be indicative of early senescence in the lower older shoots of sensitive genotypes. Overall, *GhSOS1* activity did increase with stress severity with no significant implication to reduced Na^+^ accumulation in the shoot.

Since *GhNHX1* genes function to sequester Na^+^ in the vacuole, their activity is unrelated to Na^+^ uptake but may contribute to total accumulation. In the upper and more sensitive L3 organs of the shoot (Figure 7E), *GhNHX1.A2* was upregulated in the tolerant genotypes at all stress levels. This may explain why in all tissues, the rate of Na^+^ accumulation declined except in the upper shoots. In the roots through middle organs of the shoot (R-L1-L2), *GhNHX1.D4* was upregulated in sensitive genotypes under severe salinity (Figures 7B–D). At such point in the experiment, these organs were severely compromised and activation or repression of gene expression may represent a last attempt at defense and survival.

Taken as a whole, Na^+^ transport, mobilization and sequestration, although important to the baseline tolerance, apparently explained only a relatively small proportion of the total variation for salinity tolerance across the germplasm panel, especially when the stress is pushed at the very extreme level. It appeared that Na^+^ sequestration mechanism is a highly conserved component of the adaptive mechanisms that has been conserved across landraces during domestication of *Gossypium hirsutum* which may have been favorably selected also during subsequent improvements by breeding. A more reasonable explanation for the increase in Na^+^ accumulation in the shoot could be an efficient transpiration under stress. On average, the more tolerant genotypes had 30 mmol/(m^2^⋅s) greater stomatal conductance than sensitive genotypes. Overall, based on the current results, evidence supporting that exclusion or avoidance is the most critical mechanism that could fully explain the total variance for salinity tolerance potential across the cultivated *Gossypium* could not be substantiated.

### Differential Contributions of A and D Subgenomes

Polyploidy can have a positive effect on abiotic stress tolerance, and this phenomenon has been documented by seminal observations in bread and durum wheats (Munns et al., 2012). Diploid D-genome species such as *Gossypium davidsonii* are known to be salt-tolerant (Zhang et al., 2016). It has been proposed that domestication of *G. hirsutum* favored the orthologs of the D-subgenome for abiotic stress tolerance mechanisms (Zhang et al., 2015). Comparison of the effects of salt stress on the differential regulation of orthologous *GhHKT1*, *GhSOS1*, and *GhNHX1* from the sub-genomes of the tetraploid *G. hirsutum* did not show a clear indication that either subgenome exerts a more dominant contribution than the other. Expression of *GhHKT1* tend to always favor the A-subgenome orthologs. On the other hand, expression of *GhSOS1* tend to favor the D-subgenome orthologs when taking into consideration that *GhSOS1.A12* while located in the A-subgenome originated from the D-subgenome. Additionally, expression of *GhNHX1* showed different spatio-temporal patterns, *i.e.*, the D-subgenome orthologs were dominant at severe stress in inferior genotypes, while the A-subgenome orthologs were dominant in superior genotypes particularly in L3 organs (Figure 7E).

Perhaps more intriguing was the relationship of the orthologous genes to their progenitors and their positions in the A and D subgenomes. While *GhHKT1* has two loci corresponding to each subgenome, *GhSOS1* has a duplicate copy of the *G. raimondii* ortholog in *G. hirsutum* on chromosome-12A. *GhNHX1* is another example of the dynamic nature of polyploidy in the sense that *G. raimondii* has three copies, whereas *G. arboreum* only has two. Combining the genomes, we would expect *G. hirsutum* to have five, but instead, there are only three. The *G. raimondii* ortholog that would correspond to the gene copy on chromosome-7 and the *G. arboreum* gene that corresponds to the paralog on chromosome-8 have been lost to maintain the gene copy number similar to the D-genome parent.

### Significance of Physiological Networks

At moderate to severe salinity, we have observed the clear onset of injury in the oldest L1 leaves in the inferior genotypes first. As stress continued over time with increasing intensity, the injury progressed through the vertical shoot axis toward L2 and L3. Similar patterns of injury started appearing in tolerant genotypes but with significant time delay. It was this observation that led to the central hypothesis that Na^+^ toxicity in the upper portion of the plants was averted by the differential activity of Na^+^ transport system, which acted to reduce or sequester Na^+^ in the more sensitive organs of tolerant genotypes. As the data revealed, genes involved in Na^+^ homeostasis exhibited some patterns of genotype-specific and vertical axis position-specific expression, but these patterns alone did not translate to differential spatio-temporal Na^+^ accumulation. Instead, the differential responses observed that represent total phenotypic variance across the minimal comparative panel appeared to be consequences of complex physiological and biochemical interactions.

Of the physiological parameters compared across the comparative panel, total antioxidant capacity, PRX, stomatal conductance, chlorophyll content and divalent cation content clearly explained more of the variance in SES. Na^+^ or its transport appeared to have mixed significance. Within each physiological class, *Ion Transport and Homeostasis*, *Photosynthesis and Metabolism*, or *Radical Scavenging and Oxidative Defenses*, only the model of *Photosynthesis and Metabolism* in shoots fit the data. This suggests much of the unexplained variance is in latent, unmeasured variables. Our models also assumed SES to be an endogenous variable dependent on the outcomes of measured variables, but this relationship could go the either way. For example, higher antioxidant activity did not improve salt tolerance, but instead, healthier plant produced more antioxidants.

The general trends revealed by *randomForest* indicated that the *first-line-of-defense* including radical scavenging and Na^+^ efflux and homeostasis contributed to the overall stress tolerance potential across genotypes. However, the relative importance of such mechanisms tended to decline with prolonged exposure to stress and with greater severity of cellular toxicity. Based on these models, there appears to be a much more complex synergy at the cellular, biochemical and molecular levels beyond the *first-line-of defense* that could provide a better quantitative measure of the total phenotypic variance. Much of what we have revealed in this study are the components of the baseline tolerance mechanisms that contribute to the inherently high salinity tolerance potential of *Gossypium hirsutum* relative to most other crop plants. Although some of the variations observed was explained by the multi-dimensional data, the major sources of variation were still not fully accounted for, indicating that there is an additional layer contributing to the variance yet to be uncovered. While this study leaves questions yet to be answered, understanding the foundations for the baseline tolerance is important because it becomes the basis for interpreting the more extensive transcriptome analysis that is currently underway. The emerging paradigm associated with the Omnigenic Theory for quantitative traits that is changing the perspective on complex traits in humans should provide a good backbone and inspiration to examine the major physiological and biochemical components (core effects) and the multitude of peripheral or trans-effect components that account for the total adaptive potential of a given genotype (Boyle et al., 2017).

## Data Availability Statement

The datasets presented in this study can be found in online repositories. The names of the repository/repositories and accession number(s) can be found in the article/ Supplementary Material.

## Author Contributions

KC performed the research, designed the experiments, analyzed the data, and co-wrote the manuscript with BR. IP contributed to the gene expression experiments. LH assisted in germplasm selection from the GDRS. MS assisted in the design of physiological experiments. BR conceptualized the whole project, designed the experiments, and wrote the manuscript. All authors contributed to the article and approved the submitted version.

## Conflict of Interest

MS was employed by BASF Corporation. The remaining authors declare that the research was conducted in the absence of any commercial or financial relationships that could be construed as a potential conflict of interest.

## Supplementary Material

Supplementary Figure 1 Boxplots of dry weights from the initial optimization experiments. **(A)**, total dry weight from plants after one week with an input 200 mM NaCl (final solution of EC∼15dS/m) followed by an input of 300 mM NaCl (final solution of EC∼25dS/m). Variation was significant (ANOVA, F = 2.7, *p* = 0.0296, groups assigned by Tukey pairwise comparisons). **(B)**, total dry weight compared to control. Overall, the model is significant, but only line 7 which did not acclimate well to hydroponics. Otherwise, there were no significant differences when stressed plants were compared to their controls (ANOVA, F = 4.255, *p* = 0.0027, groups assigned by Tukey pairwise comparisons).

Supplementary Figure 2 Boxplots of SES for the expanded, screened *GDRS* accessions. Our minimal comparative panel are colored (Groups assigned by Tukey pairwise comparisons).

Supplementary Table 1 qRT-PCR conditions and primers.

Supplementary Table 2 Path Analysis Vectors and References.

Supplementary Dataset 1 Raw Phenotypic Data. Available at: https://doi.org/10.5061/dryad.6wwpzgmwd.

Supplementary Dataset 2 *HKT1* Gene Alignment.

Supplementary Dataset 3 *SOS1* Gene Alignment.

Supplementary Dataset 4 *NHX1* Gene Alignment.
