# Supplementary figures and images for: Networks of Physiological Adjustments and Defenses, and Their Synergy With Sodium (Na+) Homeostasis Explain the Hidden Variation for Salinity Tolerance Across the Cultivated Gossypium hirsutum Germplasm
Source: Front Plant Sci. 2020 Dec 8;11:588854. doi: 10.3389/fpls.2020.588854 (PMC7752944; doi:10.3389/fpls.2020.588854)

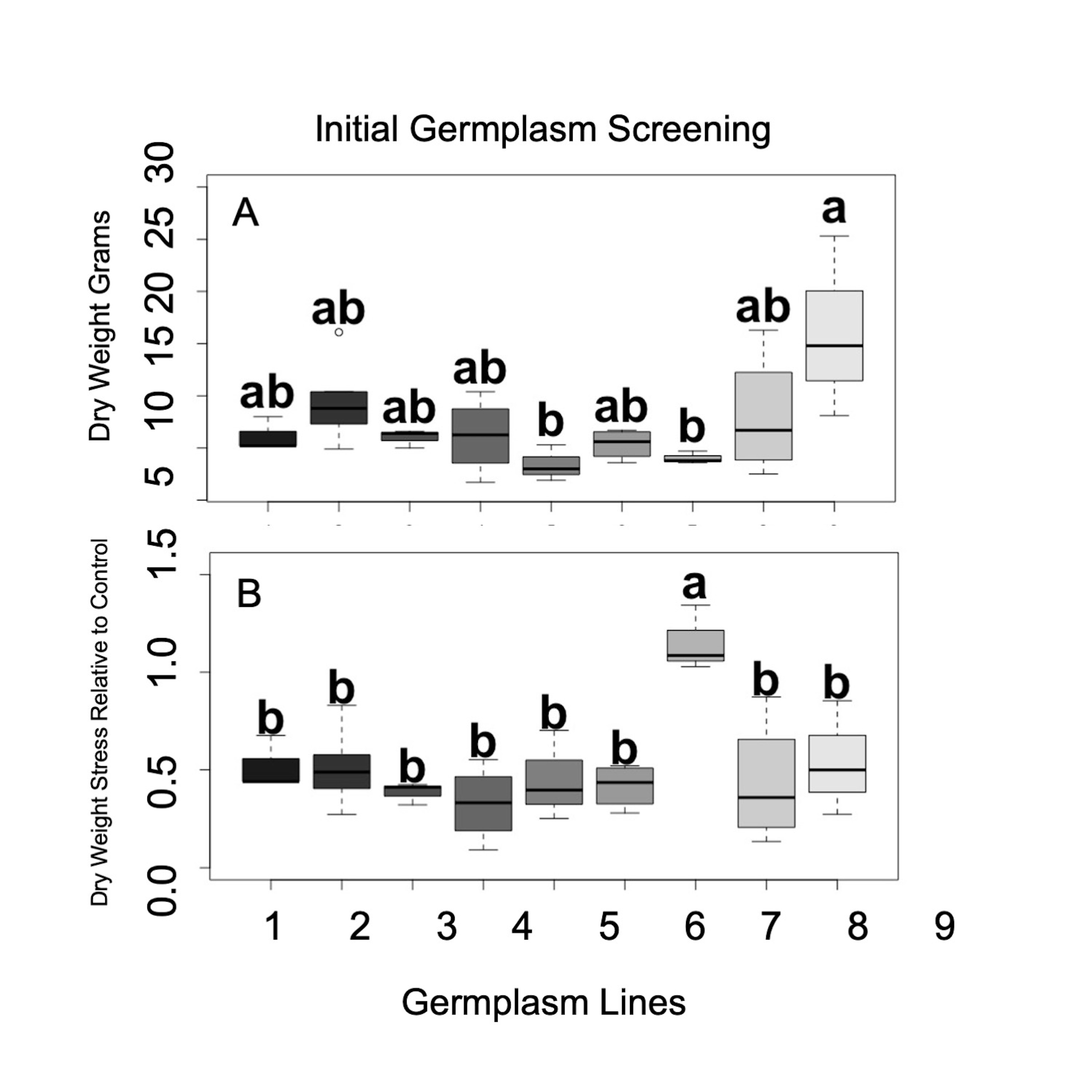

Supplement: Supplementary Figure 1 — Boxplots of dry weights from the initial optimization experiments. (A), total dry weight from plants after one week with an input 200 mM NaCl (final solution of EC∼15dS/m) followed by an input of 300 mM NaCl (final solution of EC∼25dS/m). Variation was significant (ANOVA, F = 2.7, p = 0.0296, groups assigned by Tukey pairwise comparisons). (B), total dry weight compared to control. Overall, the model is significant, but only line 7 which did not acclimate well to hydroponics. Otherwise, there were no significant differences when stressed plants were compared to their controls (ANOVA, F = 4.255, p = 0.0027, groups assigned by Tukey pairwise comparisons). [file Image_1.jpeg]

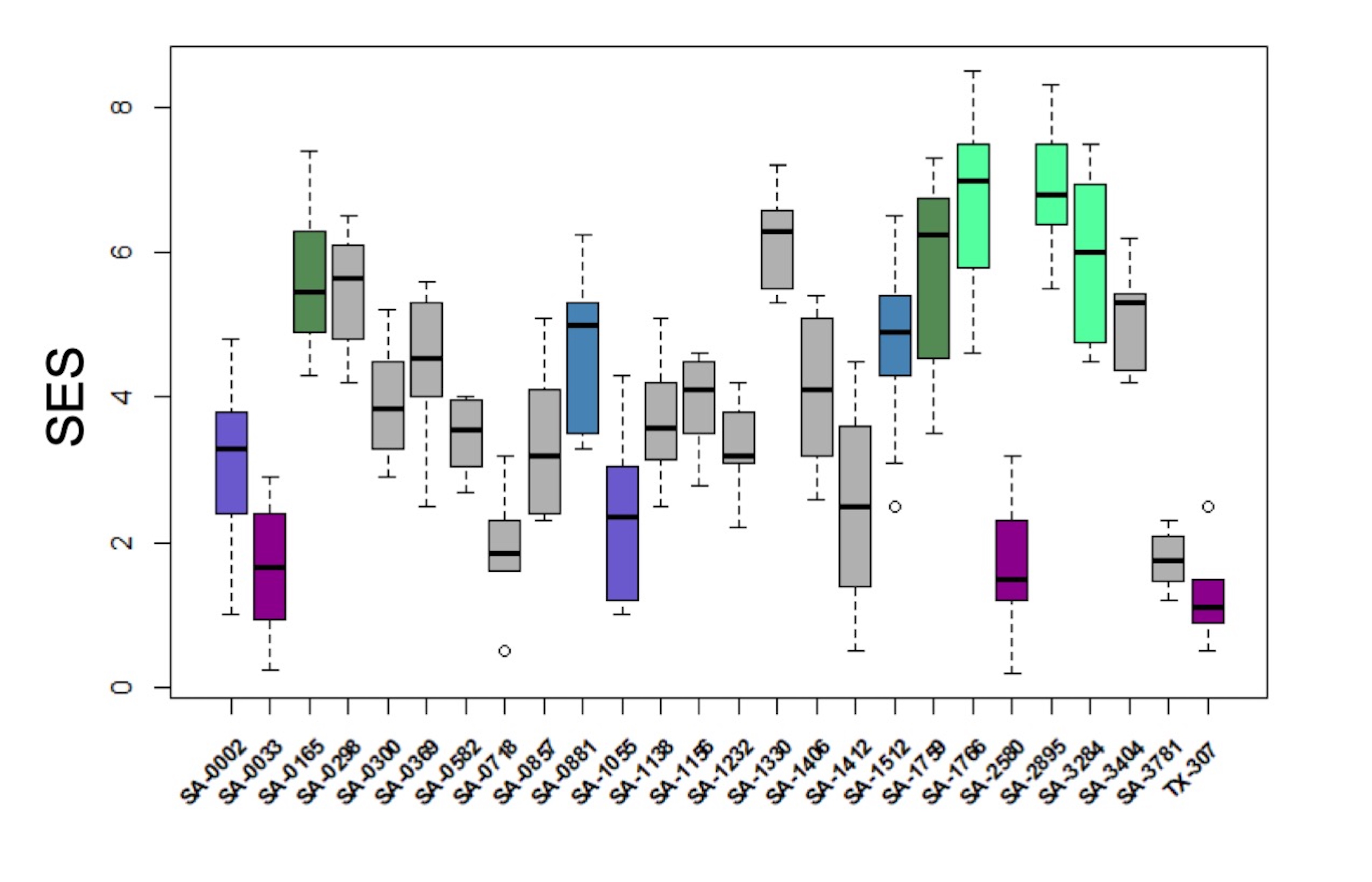

Supplement: Supplementary Figure 2 — Boxplots of SES for the expanded, screened GDRS accessions. Our minimal comparative panel are colored (Groups assigned by Tukey pairwise comparisons). [file Image_2.jpeg]
