# Supplementary material for: Networks of Physiological Adjustments and Defenses, and Their Synergy With Sodium (Na+) Homeostasis Explain the Hidden Variation for Salinity Tolerance Across the Cultivated Gossypium hirsutum Germplasm
Source: Front Plant Sci. 2020 Dec 8;11:588854. doi: 10.3389/fpls.2020.588854 (PMC7752944; doi:10.3389/fpls.2020.588854)
Supplement: Supplementary Table 1 — qRT-PCR conditions and primers. [file Table_1.DOCX]

**Supplementary Table S1.** RT-qPCR primers and conditions. The initial incubation was at 95 °C for 2 min followed by 40 cycles of 95 °C for 5 sec and 55 °C for 15 sec. Primers for reference genes were GhACT4 F, GhACT4 R, GhUBQ14 F, and GhUBQ14 R (Artico et al., 2010).

| Gene | Direction | Sequence 5' → 3' | Tm (°C) | Length (bp) |
| --- | --- | --- | --- | --- |
| *GhHKT1.1A* | Forward | ATATACCCCTACTGCTCAAAG | 54.2 | 166 |
|  | Reverse | AGTGGATATCAAACCACGAT | 54.1 |  |
| *GhHKT1.3D* | Forward | GATTGAAAGGTAAGCAGTGG | 54.3 | 191 |
|  | Reverse | TGCTCGATATTATGCGGTTT | 55.3 |  |
| *GhSOS1.6A* | Forward | GTCCGCGAATCACTAGAATG | 53.2 | 133 |
|  | Reverse | CTCTTACTCTTCCACTCTTGA | 51.0 |  |
| *GhSOS1.12A* | Forward | CATTCCACCAGGCTTCTTAAA | 52.9 | 109 |
|  | Reverse | CACATAAAAGACACTATAAGCAAT | 49.3 |  |
| *GhSOS1.7D* | Forward | TACGTGTTACCTTCGAGA | 52.1 | 102 |
|  | Reverse | AGCGATTCCTAATACCAAAGA | 54.4 |  |
| *GhNHX1.2A* | Forward | AGATTTGTCAGTGATAGCCC | 52.0 | 131 |
|  | Reverse | GACAGGGGAAACACAAAAG | 51.4 |  |
| *GhNHX1.2D* | Forward | ATGGAGAGTAAATGATCGGC | 52.1 | 100 |
|  | Reverse | TGATAGGGGAAACACGAAAG | 52.1 |  |
| *GhNHX1.4D* | Forward | GAATCAATTACTGCCCTTGC | 51.9 | 91 |
|  | Reverse | TGAAGACAAGAAGACGTGAG | 51.9 |  |

**Supplementary Table S2:** Vector descriptions and referenced studies highlighting the relationship of the interacting variables. Character interaction in bold are purportedly positive.

| Description and Path | References |
| --- | --- |
| Ion Transport and Homeostasis | |
| Ca^2+^ -> SES (a) | Zhang et al., 2014 |
| Ca^2+^ -> SOS1 (b) | Halfter et al., 2000; Chinnusamy et al., 2004 |
| SOS1 -> NHX1 (c) | Kong et al. 2016; Kong et al., 2017; Wang et al., 2017 |
| NHX1 -> Na^+^ (d) | Aspe et al., 1999; Wu et al., 2004 |
| SOS1 -> Na^+^ (e) | Halfter et al., 2000 |
| Na^+^ -> SES (f) | Leide and Saiz, 1997; Horie et al., 2009; Munns et al., 2012 |
| NHX1 -> K^+^ (g) | Bassil et al., 2011 |
| HKT1 -> Na^+^ (h) | Berthomieu et al., 2003; Davenport et al., 2007 |
| Mg^2+^ -> HKT1 (i) | Chen et al., 2017 |
| HKT1 -> K^+^ (j) | He et al., 2016; Wang et al. 2017 |
| Na^+^ -> K^+^ (k) | Shabala et al., 2010 |
| K^+^ -> SES (l) | Hauser and Horie, 2010 |
| Mg^2+^ -> SES (m) | Chen et al., 2017 |
| Photosynthesis and Metabolism | |
| DW -> SES (n) | Basal et al., 2006 |
| Cond -> DW (o) | Zhang et al., 2014 |
| Cond -> SES (p) | Yeo et al., 1991 |
| Cp -> SES (q) | Zhang et al., 2014 |
| Cp -> DW (r) | Zhang et al., 2014 |
| Pro -> SES (s) | Bojórquez-Quintal et al., 2014; Theerawitaya et al., 2019 |
| Radical Scavenging and Defenses | |
| ELI -> SES (t) | Jambunathan, 2010 |
| LP -> ELI (u) | Jambunathan, 2010 |
| PRX -> SES (v) | Gossett et al., 1994 |
| PRX -> LP (w) | Zhang et al., 2014 |
| DPPH -> PRX (x) | Gossett et al., 1994 |
| DPPH -> SES (y) | Gossett et al., 1994 |
| PER -> DPPH (z) | Gossett et al., 1994 |
| CAT <-> PER (A) | Gossett et al., 1994 |
| CAT -> DPPH (B) | Gossett et al., 1994 |
